# Investigation of enhanced adhesion of HEK293T cells on SAM-modified ITO surfaces using NMR metabolomics

**DOI:** 10.3389/fbioe.2025.1652675

**Published:** 2025-09-25

**Authors:** Oluseyi V. Ochima, Oreoluwa Alonge, Julie P. Pollak, Bo Wang, Debasish Kuila

**Affiliations:** ^1^ Department of Chemistry, North Carolina AandT State University, Greensboro, NC, United States; ^2^ Department of Chemistry and Chemical Engineering, Florida Institute of Technology, Melbourne, FL, United States; ^3^ Department of Biomedical and Chemical Engineering and Sciences, Florida Institute of Technology, Melbourne, FL, United States

**Keywords:** HEK293T, adhesion, NMR metabolomics, cell biology, ITO-MPS SAM-coated substrate, metabolites

## Abstract

Organoids are self-organizing, three-dimensional structures that replicate the main features of an organ. It is a fast-evolving area of research in regenerative medicine and cell biology. While the primary or stem cells are adapted for most of the organoid systems, Human Embryonic Kidney (HEK) 293T cells have been used in specialized applications within organoid systems when genetic manipulation is necessary. Unfortunately, HEK293T cells often suffer from loose adherence, which limits their applications in organoid formation. To improve cellular adhesion and proliferation, and to provide insights into the pathways involved in adhesion, HEK293T cells were cultured on a glass substrate sputtered with indium tin oxide (ITO) that is covered with a self-assembled monolayer (SAM) of 3-(mercaptopropyl) trimethoxysilane (MPS) (hereafter known as ITO-MPS SAM-coated substrate), a SAM of 3-(aminopropyl) triethoxysilane (APTES), and a SAM of 1-octadecanethiol (ODT). The ITO-MPS SAM scaffold yielded the most promising results, based on cell proliferation using MTT (3-[4,5-dimethylthiazol-2-yl]-2,5-diphenyltetrazolium bromide) assays. Nuclear Magnetic Resonance (NMR) spectroscopy was used to analyze the metabolomics present in the media with and without ITO-MPS-SAM coated substrates for a period of 120 h. The findings from the MTT assays demonstrate improved cell adhesion and proliferation on the ITO-MPS SAM-scaffold. The confocal microscopy images are consistent with these findings and provide a visual confirmation of the enhanced cellular environment. The metabolomic analysis yielded twenty-six metabolites, including sixteen adhesion promoters and modulators. These findings provide valuable insights into optimizing substrate conditions for improving cell adhesion and proliferation in HEK293T cells, potentially enhancing 3D cell culture and organoid research. The study also highlights novel metabolomic changes associated with improved cellular adhesion, contributing to the broader field of regenerative medicine and tissue engineering.

## 1 Introduction

Cell adhesion and proliferation are essential in cell biology, directly affecting cell communication, tissue development, and cellular responses to environmental stimuli (Hynes, 2009). These biological processes are crucial for developing *in vitro* models that replicate *in vivo* conditions, particularly in three-dimensional culture and organoid systems (Langhans, 2018). Cell adhesion is crucial for matrix remodeling, signal transduction, preservation of tissue integrity and function, and for the anchorage-dependent proliferation of cell lines (John Jayakumar et al., 2020). Recent breakthroughs in surface chemistry, particularly functionalization of Indium tin oxide (ITO) substrates with self-assembled monolayers (SAMs), have demonstrated potential in enhancing cellular adhesion and proliferation in *in vitro* cell culture studies. More specifically, SAMs can be modified with diverse chemical groups to change the physicochemical characteristics of the substrate, affecting cellular activity. This happens through alterations in surface charge, roughness, and hydrophobicity (Ulman, 1996). ITO is a transparent and conductive substrate, which, when coated with SAMs, provides a promising platform for enhancing cell adhesion while facilitating optical and electrochemical observation of cellular activities (Y. H. Choi et al., 2015; Zhao et al., 2021).

The human embryonic kidney 293 (HEK293) cell line is a case study of weakly adherent cells. The cell line was derived from the kidney of an aborted female human fetus and was immortalized in 1973 (Liu et al., 2021). HEK293T cells on SAMs with different end groups on ITO were studied to investigate their cellular activity because HEK293T cells have demonstrated significant use in cell biology, especially in conventional 2D cultures, and are increasingly being used in organoid research and 3D culture systems (Iuchi et al., 2020; Glykofrydis et al., 2021). The other reason for our choice of HEK293T is to study metabolomics using NMR spectroscopy. HEK293T cells, which express the SV40 large T antigen, are extensively utilized in biomedical research and molecular biology due to their superior transfection efficiency, strong protein expression capabilities, and rapid proliferation (Bae et al., 2020; Mobasheri et al., 2016; Zhu, 2013). For instance, HEK293T cells are widely used for gene expression research, protein production, and viral vector development (Thomas and Smart, 2005; Kritchenkov, 2017). Furthermore, their swift proliferation facilitates the generation of substantial experimental data in a relatively short period of time, which is particularly advantageous for analyzing metabolites and high-throughput applications like drug screening (Edmondson et al., 2014).

**FIGURE 1:**
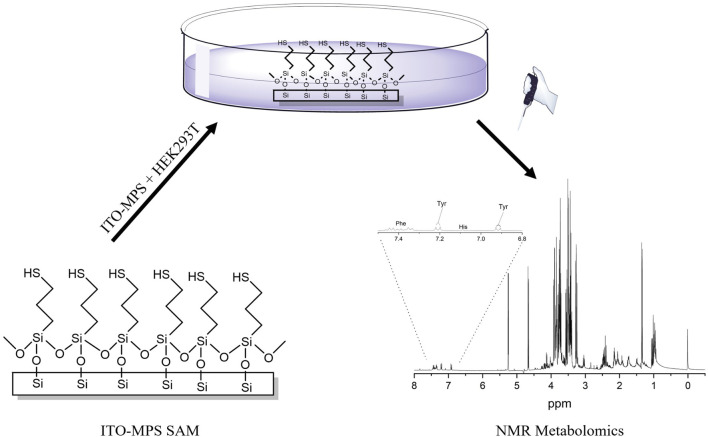
Scheme showing the ITO-MPS-SAM coated substrate, HEK293T cell culture on the ITO-MPS-SAM-coated substrate, and the NMR-based metabolomic analysis.

Metabolomics is a discipline that deals with the effects of various treatments on a large number of metabolites analyzed simultaneously using analytical platforms (Jiang et al., 2020). In the past 10 years, a sensitive and reliable analytical technique such as proton (^1^H) NMR spectroscopy has been used for a greater understanding of the metabolic changes underlying phenotypic changes (Nizioł et al., 2020; Avison et al., 1986). Metabolomics-based nuclear magnetic resonance (NMR) spectroscopy is extensively utilized for drug discovery (Wang et al., 2020; Bédard et al., 2020) and has been applied to the study of nutritional science, environmental science, and plant sciences (Murovec et al., 2018). It provides insights into the biological mechanisms and pathways associated with cell adhesion. HEK293T cells have demonstrated significant use in conventional 2D cultures, and are increasingly being used in organoid research and 3D culture systems (Iuchi et al., 2020; Glykofrydis et al., 2021). Organoids are self-organizing, three-dimensional entities that replicate essential characteristics of organs. They represent an evolving domain of research in regenerative medicine and cell biology (Clevers, 2016). Organoids are often produced from primary cells or stem cells; however, HEK293T cells have been utilized in particular applications within organoid systems, especially where genetic manipulation is necessary (Astashkina et al., 2012). HEK293T cells have also been used in hybrid models and co-culture systems, particularly in cancer and virus studies alongside stem cells or primary cells to facilitate the differentiation and growth of organoids. For example, in neuronal organoid models, HEK293T cells engineered to express particular growth factors have been utilized to promote neural differentiation and maturation, emphasizing their efficacy in developing more intricate organoid systems (Lancaster and Knoblich, 2014). These models facilitate the connection between conventional 2D systems and complex 3D systems by enabling researchers to genetically modify HEK293T cells while preserving the tissue-like architecture of organoid models. Although HEK293T cells are widely utilized, they exhibit weak adherence on standard cell culture substrates, limiting their use in more complex applications, such as organoid systems and 3D cultures, where robust cell-matrix interactions are essential (Thomas and Smart, 2005; Nizioł et al., 2020). In recent years, collagen has been used to improve cell adhesion on tissue culture substrates. However, most of the time, laboratories have poor guidance on the optimization and/or selection of collagen on these substrates (Chua and Lim, 2023). Cells need to adhere properly to promote their proliferation and the expression of their functionality (Nizioł et al., 2020). Thus, alternative strategies are needed to improve HEK293T cellular adhesion and proliferation. In this study, we explored the ability of various SAM scaffolds to enhance HEK293T adherence and subsequent proliferation. Furthermore, to gain insights into the cellular outputs of the cells, we measured their metabolomics profiles. To our knowledge, the NMR metabolomics for HEK293T cells on a SAM scaffold has not yet been reported. We show that the adhesion and proliferation of HEK293T cells can be improved by culturing the cells on a SAM scaffold, and through the NMR-based metabolomic analysis, we provide an extensive dataset that could be valuable for understanding the facilitators of adhesion and the complex pathways involved in HEK293T cellular adhesion (Figure 1).

The cell proliferation on the SAM scaffolds was measured using the MTT (3-[4,5-dimethylthiazol-2-yl]-2,5-diphenyltetrazolium bromide) assays. The findings from the MTT assay show improved cell adhesion and proliferation on the ITO-MPS SAM-coated substrate. The confocal microscopy images are consistent with these findings and give a visual confirmation of the enhanced cellular environment. NMR spectroscopy was used to analyze the metabolomics present in the media with and without ITO-MPS-SAM coated substrates for a period of 120 h. The metabolomic analysis yielded twenty-six metabolites, including sixteen promoters and modulators of adhesion. This provides new insights into the metabolomic changes linked to enhanced cellular adhesion and establishes a more effective platform for culturing HEK293T cells by enhancing their adhesion and functionality in both 2D and 3D cultures, as well as advanced organoid systems, where dependable cell-matrix interactions are essential for replicating tissue function and architecture (Lancaster and Knoblich, 2014). It advances the field of cellular biology research by presenting an improved methodology for culturing HEK293T cells.

## 2 Materials and methods

### 2.1 Substrate and SAM preparation

The ITO-coated glass slides were cleaned by sonication in toluene, acetone, and ethanol, each for 5 min, and then in deionized (DI) water for 30 min. The substrates were then rinsed with DI water and dried with nitrogen (N_2_). The remaining ITO-coated glass substrates were immersed in the neat liquid of 1-octadecanethiol (ODT) for 1 h to prepare SAMs with −CH_3_ end groups, followed by rinsing in ethanol and drying with N_2_. SAMs of the −NH_2_ and −SH end groups were prepared by immersing the ITO substrates in a 1 mM ethanolic solution of APTES and MPS for 12 h, followed by rinsing with ethanol and drying with N_2_. All the samples were sterilized in 70% ethanol for 1 day before seeding the cells (Aithal et al., 2016).

### 2.2 SAM characterization

#### 2.2.1 Contact angle measurements

Contact angle measurements for all substrates modified with SAMs were done using the sessile drop method on a Contact Angle SOP Kruss Bruker DSA25. Data Physics Instruments (DPI) contact angle goniometer (Aithal et al., 2016). A mL syringe support above the sample stage had a 0.5 L droplet of water hanging from its tip. The syringe tip was advanced towards the sample until the drop made contact with the sample surface. The syringe was then retracted, leaving the droplet on the sample. This was recorded on a charge-coupled device (CCD) camera and stored in the SCA 202 software.

#### 2.2.2 Fourier-transform infrared spectroscopy (FTIR) measurement

The reflectance absorption IR and attenuated total reflection spectra (RAIRS and ATR) of SAMs on ITO were obtained using a Smart Saga with mercury cadmium telluride (MCT) detector for RAIRS, while Smart Miracle with deuterated-triglycine sulfate (DTGS) detector for ATR. The chamber of the instrument was constantly purged with N_2_. The spectra were recorded at a resolution of 4cm^-1^ (512 scans), and the baseline was corrected using the bare ITO glass slide. The observed peaks were assigned to vibrations according to published FTIR studies of similar compounds.

#### 2.2.3 X-ray photoelectron spectroscopy (XPS) measurement

XPS was conducted using an Al monochromatic X-ray at a power of 350 W in a PHI5600S multi-technique system. Al Kα with 10 mA and 15 kV peaks were resolved by the calibration on carbon. The survey scan of the XPS spectrum was done in the binding energy range from 0 to 1,200 eV. The XPS spectra of Si 2p, O1s, Al 2p, and P 2p of the geopolymers before and after exposure were assessed from the position of binding energy lines, intensity, and percentage composition (Vanitha and Jeyalakshmi, 2023).

#### 2.2.4 Scanning electron microscopy

The morphological features of the ITO-SAM scaffold were characterized using a scanning electron microscope (SEM) (Machida et al., 2023). A Gemini 300 SEM provided a resolution of the images as low as 2 nm at 15 kV. The samples were gold-coated at a thickness of 4–7 nm (Vanitha and Jeyalakshmi, 2023).

### 2.3 Cell culture

The HEK293T cells (ATCC, Manassas, United States) were procured in cryopreserved vials and cultivated using normal protocols for mammalian cell culture. Briefly, the cells were thawed and cultured in T25 flasks (Corning, Germany) at 37 °C with 5% CO_2_ using Dulbecco’s Modified Eagle high Glucose medium (DMEM) supplemented with 10% fetal bovine serum, and 1% Penicillin-Streptomycin (10 U/mL). The cells were maintained at passage numbers between 5 and 7. Cells were split at a confluency of >85% using standard cell culture procedure (Theobald et al., 2018).

### 2.4 Cell proliferation

The MTT (3-[4,5-dimethylthiazol-2-yl]-2,5-diphenyltetrazolium bromide) assay kit was used to determine the cell proliferation after 24, 48, 72, 96, and 120 h on the various substrates. This analysis is based on the principle of measuring the activity of living cells by monitoring mitochondrial dehydrogenase activity. The density of the seeded HEK293T cells was 50,000 cells per substrate. A standard curve was established to measure MTT activity as a function of cell density. The results were presented as the average of triplicate experiments (± standard error). An increase in cell number results in an increase in the amount of formazan formed and an increase in absorbance at 570 nm (Aithal et al., 2016). The best substrate in terms of promotion of cell adherence and proliferation (ITO-MPS SAM-coated substrate) was first characterized by Fourier-Transform Infrared Spectroscopy (FTIR), contact angle measurement, scanning electron microscopy (SEM), and X-ray photoelectron spectroscopy (XPS) techniques.

### 2.5 Sample preparation for NMR experiments

The HEK293T cells were grown in a 24-well plate containing SAM-coated substrates, including Indium Tin Oxide (ITO), (3-Mercaptopropyl) Trimethoxy Silane modified Indium Tin Oxide (ITO-MPS), (3-Mercaptopropyl) Trimethoxy Silane (MPS), and Tissue culture Plastic (TCP), respectively. The cells were grown for 5 days, and samples were collected every day for the 5 days. 700 μL of the sample were collected for NMR analysis, and 100 μL were collected for the MTT assay. The NMR samples were centrifuged for 10 min at 14.8 × 10^3^ rpm. After that, the supernatant was extracted, transferred to a different microcentrifuge tube, and kept in the vacuum dryer for 7 hours. After the sample was dried, it was then mixed with phosphate buffer in which the final solution contained 90% D_2_O, 0.05% sodium azide, and 0.1 mM trimethylsilyl propionate (TSP). A volume of 600 μL of the solution was filled into 5 mm NMR tubes for NMR experiments.

### 2.6 NMR experiments and data interpretation

The NMR metabolomics analysis of media used to culture HEK293T cells for 5 days was done to study the metabolomics of HEK293T cells cultured on (3-Mercaptopropyl) Trimethoxy Silane (control), normal tissue culture plastic, Indium Tin Oxide (ITO), and (3-Mercaptopropyl) Trimethoxy Silane modified Indium Tin Oxide (ITO-MPS SAM-coated substrate) respectively. Five days were selected based on the MTT results. The experiments were done in six replicates to ensure reproducibility. The NMR studies were performed using a Bruker Ascend 400 MHz high-resolution NMR. A Sample Xpress autosampler was applied in this study, and all the experiments were carried out using ICON-NMR software (Bruker Biospin) and controlled by ICON-NMR. All the experiments were carried out after proper shimming. For the 1D NMR study, a 1D NOESY experiment with water suppression (Bruker pulse sequence name: noesygppr1d) was carried out with 32k data points, and 128 transients, which took around 11 min per sample. The 1D NMR spectra were mainly analyzed in Topspin 4.06 (Bruker Biospin). The NMR peaks were identified using Chenomx 8.6 by the chemical shifts (Chenomx Inc).

## 3 Results and discussion

### 3.1 Characterization of the ITO-MPS SAM-coated substrate

We investigated the growth of the HEK293T cells on the ITO, ITO-MPS SAM coated substrate, ITO-APTES, and ITO-ODT substrates for 12 days using the MTT assay to evaluate the cell viability and proliferation. In addition, we carried out Bradford assays to measure the total protein produced on each substrate. We observed an increased adherence of the cells on the ITO-MPS SAM-coated substrate, as shown in Supplementary Figures S2, S3, respectively. The results were visually confirmed by confocal microscopy images taken after 120 h of culture on the four substrates, as displayed in Supplementary Figure S4. We then streamlined the experiment and metabolomic analysis to focus on the study of the adherence of the cells on ITO and ITO-MPS substrate, MPS solution (Control), and Tissue culture plastic (TCP). This was done to further confirm that indeed the ITO-MPS SAM, not the ITO alone or the MPS alone, was responsible for this improved adhesion and proliferation, and to study the metabolites and the rationale involved in this enhancement. The characterization of ITO-APTES and ITO-ODT was done previously by Aithal et al.; however, the scanning electron microscopy (SEM) characterization was not done before. We have reported the SEM images in Figure 4. Furthermore, as our initial studies showed better adhesion of HEK293T cells on ITO-MPS SAM-coated substrate, we did not pursue extensive characterizations of other ITO-SAMs in this study. The contact angle and FTIR characterizations of the ITO-ODT and ITO-APTES were reported previously by Aithal et al. (2007).

#### 3.1.1 Contact angle measurement

**FIGURE 2:**
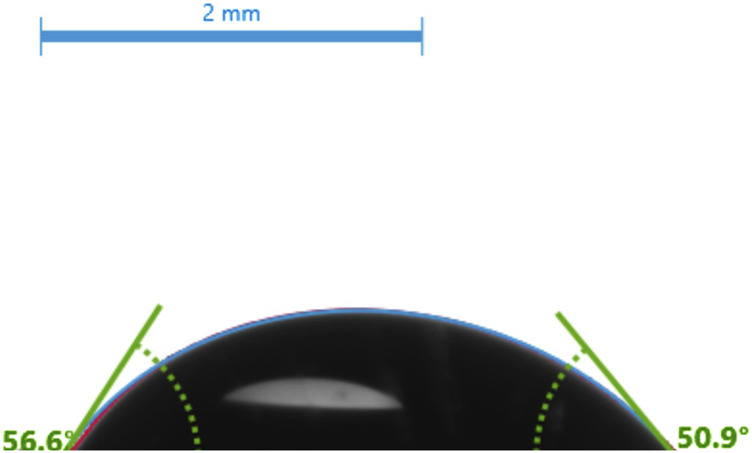
The contact angles of the ITO-MPS SAM-modified surface.

The contact angle measurement provides the hydrophobicity and hydrophilicity of our SAMs. Figure 2 shows the ITO-MPS SAM-modified surfaces’ contact angles. The glass substrate modified with MPS (−SH) had a contact angle of 54° ± 3, showing a behavior that is intermediate between hydrophilic APTES and hydrophobic ODT self-assembled monolayer (Aithal et al., 2007). The hydrophilicity of the SAM substrate contributes to the cell adhesion and proliferation of the cells in an aqueous environment under standard conditions. The hydrophilicity of a SAM has a considerable impact on its capacity to promote cell adhesion. Hydrophilic surfaces have a strong tendency to absorb proteins from.

The cell culture medium (Ulman, 2013). The adsorbed proteins, such as vitronectin and fibronectin, act as vital attachment points for cell surface integrins, which in turn help in cell adhesion.

#### 3.1.2 Fourier-Transform Infrared Spectroscopy/attenuated total reflectance (FTIR/ATR)

The FTIR of SAMs has been reported elsewhere (Aithal et al., 2007). As the major focus of the current studies is the ITO-MPS SAM-coated substrate due to higher proliferation of cells, we do not discuss other SAMs further. Some of the structural groups present on the ITO-MPS substrate, characterized using FTIR/ATR spectroscopy, are provided in Table 1. The FTIR/ATR spectrum for ITO-MPS on a glass substrate is shown in Supplementary Figure S1. The medium intensity C-H bands are observed at 2850cm^−1^, and 2,920 cm^−1^, respectively. The peaks due to CO_2_

**TABLE 1:** The Intensity and Peak Positions of the structural groups present in the FTIR/ATR spectrum of ITO-MPS SAM coated on a glass substrate.

Structural group	Intensity peak position (cm^−1^)	Peak mode	Peak description
C-O	1,583	Stretching	Medium
S-H	2,608	Stretching	Very weak
C-H	2,850 and 2,920	Stretching	Medium
O-H	3,649 and 3,700	Stretching	Medium sharp

Could not be removed completely. Medium sharp O-H bands are observed at 3,649 cm and 3,700 cm^−1^. The very weak peak at 2,550 cm^−1^, observed in the ATR spectrum of MPS on ITO, corresponds to the thiol S–H stretching. This finding is indicative of the ITO-MPS monolayer with the respective end groups.

#### 3.1.3 X-ray photoelectron spectroscopy (XPS)

**FIGURE 3:**
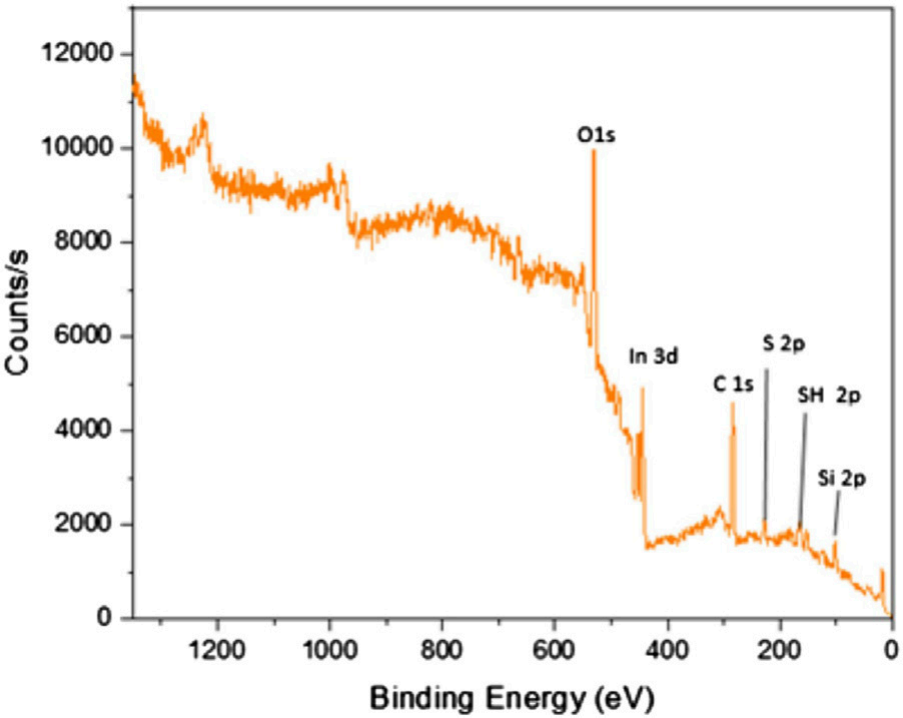
X-ray photoelectron spectroscopy analysis of ITO-MPS substrate.

The X-ray photoelectron spectroscopy (XPS) was used for analyzing the surface properties of the ITO-MPS scaffold to measure elemental composition as well as the electronic and chemical state of the atoms in the ITO-MPS substrate. Carbon materials have fairly diverse binding energies (Chen et al., 2020). Figure 3 shows that the ITO-MPS substrate contains oxygen, indium, carbon, sulfur, thiol, and silicon at 1s, 3d, 1s, 2p, 2p, and 2p electronic states, respectively. The carbon to carbon (C-C) bonds, and carbon to oxygen (C-O) bonds were also observed in the ITO-MPS SAM. The presence of In_2_O_3,_ SnO_2,_ SnO peaks were observed at 444.5eV, 486.6eV, 486.0eV, respectively. Sulphur and silica were also observed, accounting for the presence of siloxane.

#### 3.1.4 Scanning electron microscopy (SEM)

**FIGURE 4:**
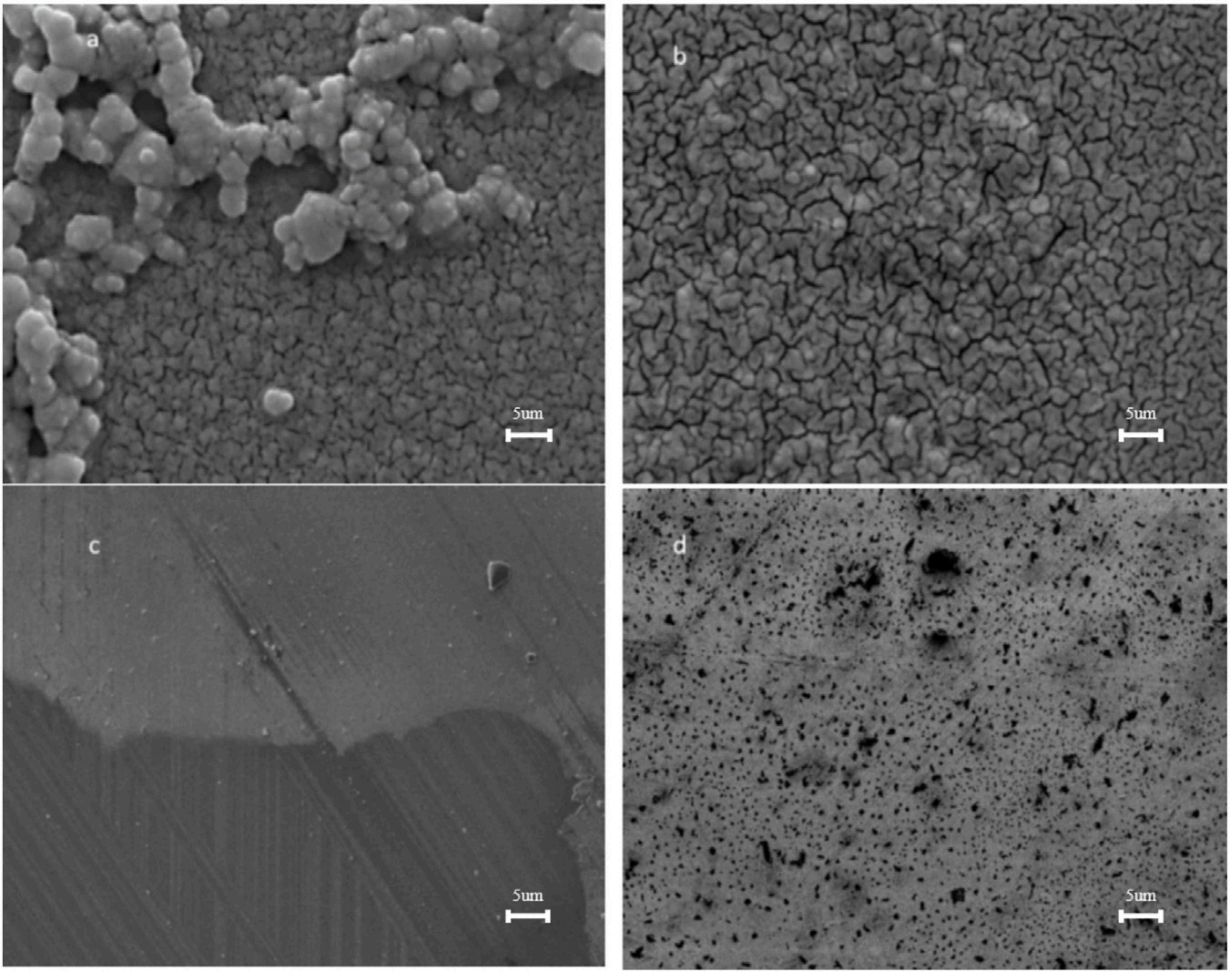
SEM images of **(a)**. ITO-MPS **(b)**. ITO **(c)**. ITO-APTES and **(d)**. ITO-ODT (10x).

The SEM images and particle size distributions of the ITO-MPS SAM are shown in Figure 4. Figure 4a shows spherical dark particles of averagely equal sizes above a somewhat folded monolayer below. This image is similar to that in Figure 4b, only that in Figure 4b, the particles are transparent, and some are folded to form the self-assembled monolayer. Compared with the microstructure needle-like particles of ITO-APTES (Figure 4c), some of which are appropriately intersected, the image of Figure 4a clearly shows little or no needle-like particles. Figure 4d shows dark spherical particles evenly distributed on the ITO-ODT SAM surface.

### 3.2 HEK293T attachment and morphology

During experiments related to the use of HEK293T cells in general cell biology, we discovered that the common problem faced by researchers working with HEK293T cells is the loose adherence of the cells, and some existing solutions to this problem either have negative effects on the cells in the long run or are expensive. We decided to test the effect of culturing HEK293T cells on several ITO-SAM-coated glass scaffolds to see if any of them would lead to an increased adhesion and proliferation of the cells when compared to the tissue culture plastic (TCP), which is the standard cell culture vessel. We initially cultured the cells on ITO, ITO-APTES, ITO-ODT, and ITO-MPS SAM-coated substrates after characterization using SEM (Figure 4). The results from these experiments showed that the ITO-MPS SAM-coated substrate yielded the best results based on MTT and Bradford assays (Supplementary Figures S2, S3). We used the Bradford total protein assay as an indicator of total cell content. We then proceeded to streamline the experiment to the culture of the HEK293T cells on the ITO-MPS SAM-coated substrate, on ITO-SAM alone, in MPS solution alone (negative control), and in TCP which is the known cell culture vessel (to further confirm the results and to do some in depth ^1^H NMR metabolomic analysis) for 120 h, during which samples were taken out on a daily basis for the MTT assays and the H^1^ NMR metabolomic analysis. The experiments were conducted in three replicates for reproducibility. These results presented the ITO-MPS SAM-coated substrate as the best in terms of promoting adhesion and proliferation of the cells, suggesting that the hydrophilicity of the ITO-MPS SAM-coated substrate contributed to the improved adhesion and proliferation of the cells (Figure 2). Confocal Microscope images of HEK 293T cells cultured on ITO-MPS with varying magnifications taken after 120 h of culture are shown in Supplementary Figure S6. The ^1^H NMR metabolomic analysis of the HEK293T cells cultured on the ITO-MPS SAM-coated substrate yielded twenty-six metabolites, including sixteen promoters and modulators of adhesion. This may explain why the ITO-MPS SAM-coated substrates had the best results in terms of promoting adhesion and proliferation of the HEK293T cells.

**FIGURE 5:**
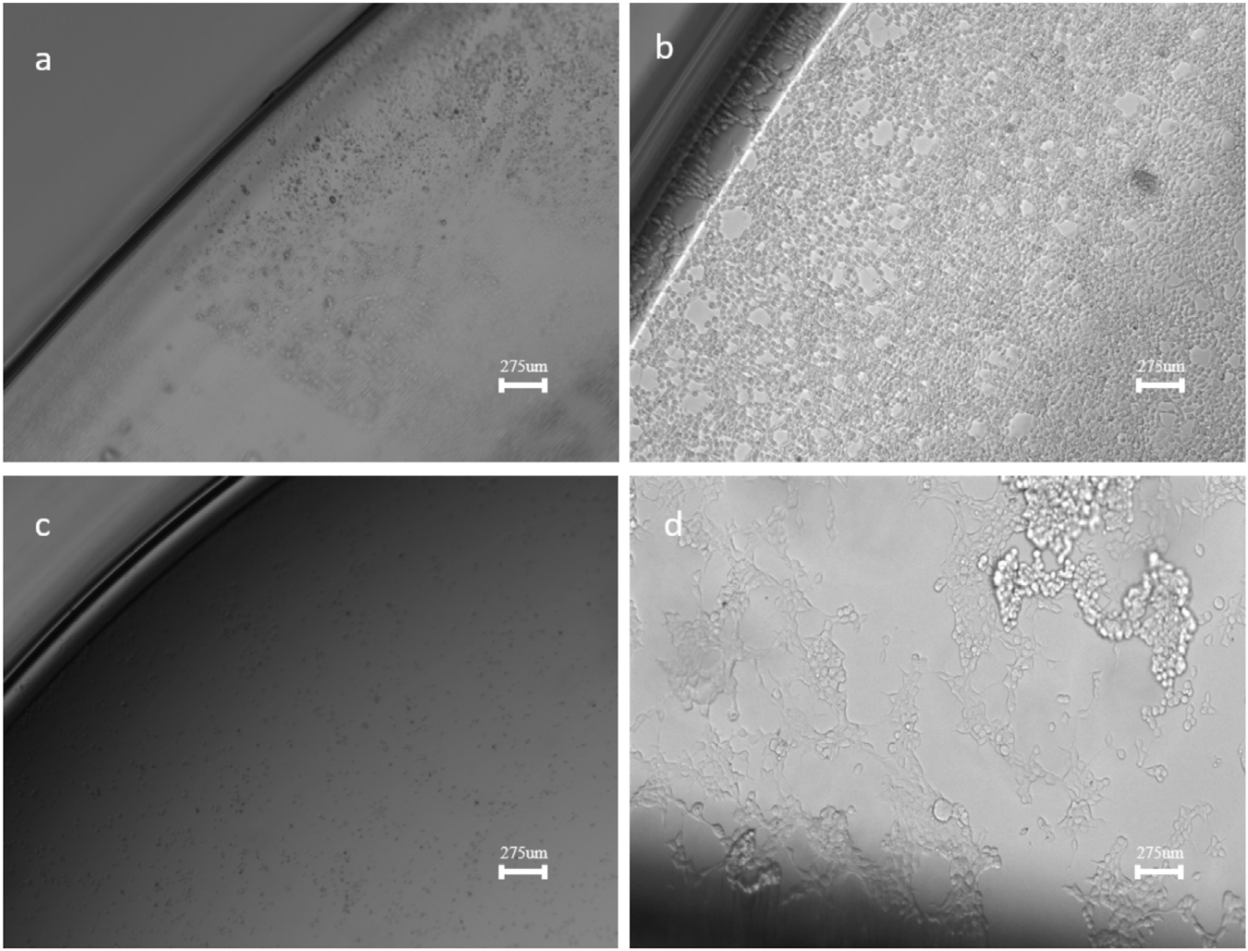
Confocal Microscope images of HEK293T cells on **(a)**. ITO **(b)**. ITO-MPS SAM-coated substrate **(c)**. MPS and **(d)**. TCP substrates (10x) after 120 h of culture.

Confocal microscopy was used to observe the adherence and morphology of HEK293T cells on different surfaces. Figure 5 and Supplementary Figure S5 show the confocal microscope images of HEK293T cells cultured on bare ITO, ITO-MPS, MPS, and TCPs after 120 h. One of the objectives of our study is to investigate the effect of terminating groups of SAMs on cellular adhesion. In the case of ITO-MPS, the tethering of the -SH groups to the hydrophobic areas of the surface of the kidney cells, along with the release of many water molecules, may be responsible for the attachment of the kidney cells. Thiol (-SH) groups have the.

Ability to participate in hydrogen bonding, which enhances the interactions between components of the extracellular matrix and adhesion molecules on the cell surface. In addition, the -SH groups can participate in the formation of disulfide bonds, which establish stronger linkages between intracellular proteins and extracellular matrix proteins, potentially promoting adhesion. Cells cultured on different substrates show round shapes, with some cells transformed into a polygonal shape after 24 h, especially on the ITO-MPS SAM. The bare ITO (Figure 5a) supports the attachment of HEK293T cells and proliferation; however, surface modification in ITO-MPS (Figure 5b) yields higher cell growth at physiological conditions. The cells on the ITO substrate still appear somewhat rounded after 24 h and smaller in size, but the ITO-MPS substrate has an epithelial-like morphology characterized by an elongated, flattened shape with distinct boundaries. In the MPS solution (Figure 5c), no live cells were observed at the end of the experiment, suggesting the occurrence of cell death on this substrate. The cells cultured on the standard TCP (Figure 5d) also had a somewhat elongated morphology, but most of the cells appeared to be lifted and dying after 120 h. The ability of the cells to remain well adhered, grow, and proliferate for long hours is a vital feature needed in organoid research applications, where the organoids may, for example, undergo treatment and be cultured for long hours. Cell spreading has been shown to increase with increasing substrate stiffness, and our experiment shows that HEK293T cells cultured on surfaces with ITO-MPS SAMs can result in significantly better adhesion and spreading of the cells.

### 3.3 HEK293T cell proliferation

**FIGURE 6:**
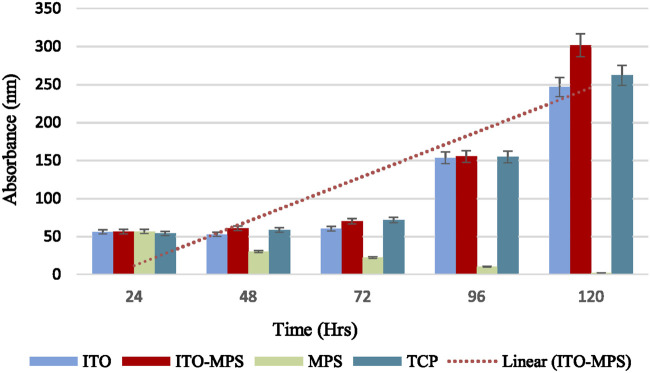
MTT assays showing proliferation of HEK293T cells cultured on various substrates for a total period of 120 h. Data shown as mean optical density (OD) of triplicate wells.

**FIGURE 7:**
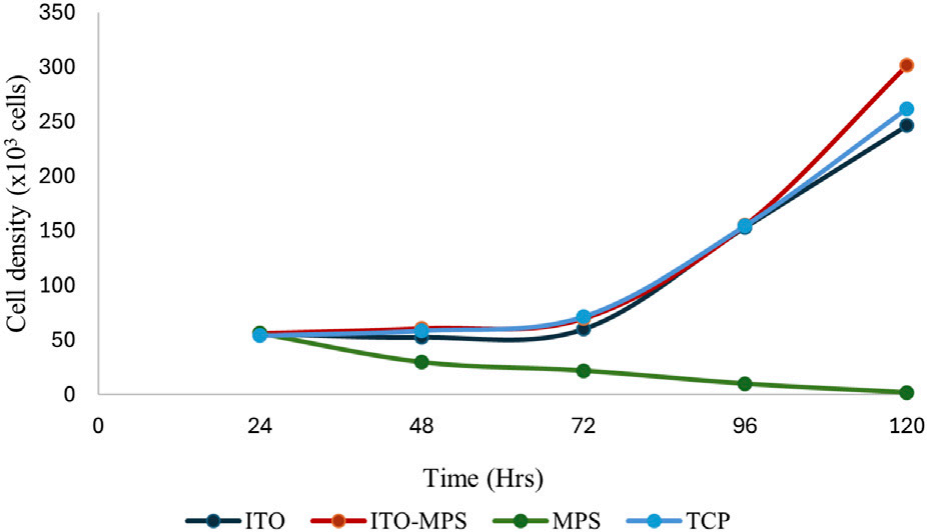
Cell densities of HEK293T cells cultured on MPS, TCP, ITO, and ITO-MPS SAM-coated substrates. Data shown as mean OD of triplicate wells.

HEK293T cell viability was also quantitatively evaluated using MTT assays. First, 50,000 cells per substrate were seeded. The cells were then cultured under standard humidified conditions, and their proliferation capacity and viability were evaluated every 24 h for the total duration of 120 h. A plot of cell absorbance on SAM-modified ITO substrates against culture duration is presented in Figure 6. A statistically significant increase in absorbance is observed on ITO-MPS SAM-coated substrate compared to MPS and ITO, showing that the substrate best supports the proliferation of HEK293T cells. To find the cell densities, the mean of corresponding absorbances (the average of three values) was compared to a standard curve (Figure 7). After 48 h, a higher cell density was observed on ITO-MPS compared to ITO, TCP, and MPS; however, the difference was not significant. Higher cell densities were also observed on ITO-MPS after a 96-h period, during which the cell density rose quickly with an increase in culture time. After 120 h, the cell density increased rapidly with an increase in culture time, with the highest cell densities observed on ITO-MPS. A significant increase in cells is.

Observed on ITO-MPS compared to MPS and ITO (p < 0.05). The cells proliferated on these substrates since the initial cell density was 50,000, and the cell densities were greater than 50,000 after 24 and 120 h. However, the MPS solution alone did not support adhesion and proliferation of the cells. There was a drastic decline in cell numbers as the days went by, and by the fifth day, there were almost no cells in the MPS-only culture vessel. Thus, the ITO-MPS SAM-coated substrate plays a significant role in adhesion and proliferation of HEK293T cells. The experiments were done in three replicates for reproducibility for a period of 120 h.

### 3.4 NMR data analysis and pretreatment

The samples were collected at the respective time intervals and pretreated by centrifuging and vacuum drying prior to NMR studies. The NMR experiment was performed, and after data acquisition, all the spectra were phased and referenced using Topsin 4.11 (Bruker Biospin, LLC). The 1D spectra were processed using Amix 4.0 (Bruker Biospin, LLC) to obtain the peak information. Using Chenomx 8.6 (Chenomx Inc) and 27 metabolite peak ranges were significantly expressed and selected from the 1D NMR analysis (Supplementary Table S1).

### 3.5 Metabolomics and analysis by ^1^H NMR

**FIGURE 8:**
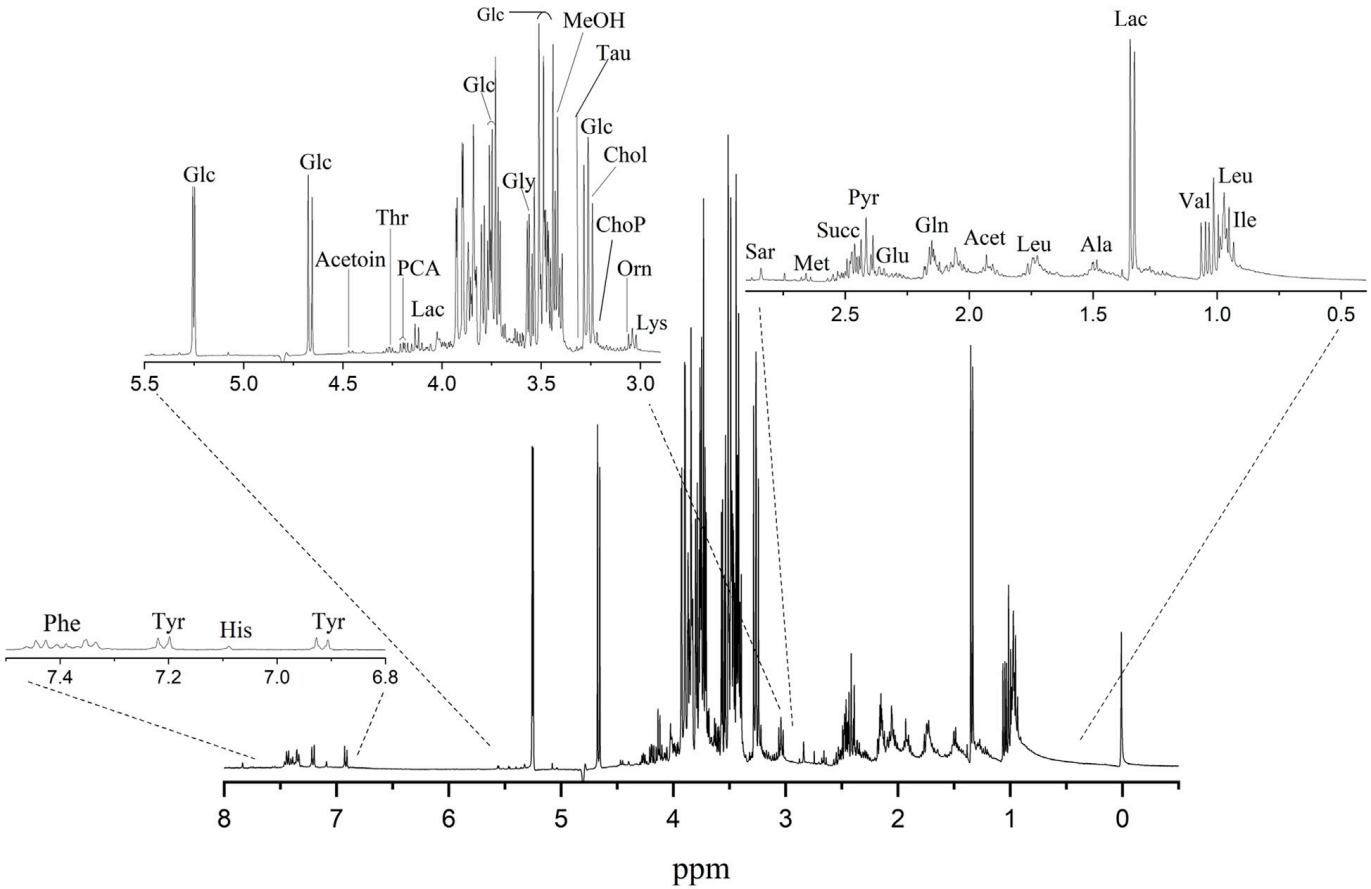
A typical 400 MHz ^1^H NMR spectrum showing the metabolites found in the aqueous extracts of HEK293T cells on ITO-MPS SAM-coated substrate. Compound names represent the predominant metabolites in the NMR spectra. 3-Letter codes are used for amino acids; Chol: choline; Glc: glucose; Lac: lactate; Orn: ornithine; ChoP: O-Phosphocholine; MeOH: methanol; PCA: pyroglutamate; Lac: lactate; Acet: acetate; Pyr: pyruvate.

In an effort to develop specific metabolomics signatures for HEK293T, the metabolic profiles of the aqueous extract of HEK293T were investigated (G. D. Brown et al., 2018). For this reason, we recorded high-resolution 1D ^1^H NMR spectra on 120 total metabolite extracts from the aqueous extract of HEK293T. Analysis of ^1^H NMR spectra resulted in the resonance assignment and quantitation of twenty-six significantly expressed metabolites (Supplementary Table S1) found in each sample. Figure 8 shows the expanded regions of the ^1^H NMR spectrum of the metabolites found in the aqueous extracts of HEK293T SAM cell-cultured media. The spectra are presented as three expanded regions: *6.8–7.5*, *3.0–5.5*, and *0.5–2.9* ppm. Supplementary Table S1 provides a list of all the metabolites significantly expressed in the aqueous extract of HEK293T cells cultured in media based on the chemical shifts data (W-M Fan, 1996) (Ritota et al., 2010).

### 3.6 Interpretation of the NMR-based metabolomics data and the pathways involved

When the NMR metabolomic analysis of the media used to culture HEK293T cells on the ITO-MPS substrate for 5 days was completed, twenty-six metabolites were observed; sixteen of which are both modulator and promoter metabolites of adhesion. These include alanine, phosphocholine, glucose, glutamate, glutamine, glycine, histidine, isoleucine, lactate, leucine, methionine, phenylalanine, pyroglutamate, succinate, threonine, and tyrosine. These results suggest that these metabolites have a significant role to play in improving the cellular adhesion beyond the known period of 72 h. Glucose, glutamine, tyrosine, threonine, and lysine metabolites, casually influence or serve as essential modulators in augmenting HEK293T cell adhesion on ITO-MPS SAM. In contrast, acetate, choline, O-phosphocholine, glycine, lactate, methionine, sarcosine, and succinate primarily demonstrate correlative relationships, wherein their levels or activities correlate with adhesion alterations, typically as a result of broader cellular responses to the SAM.

**FIGURE 9:**
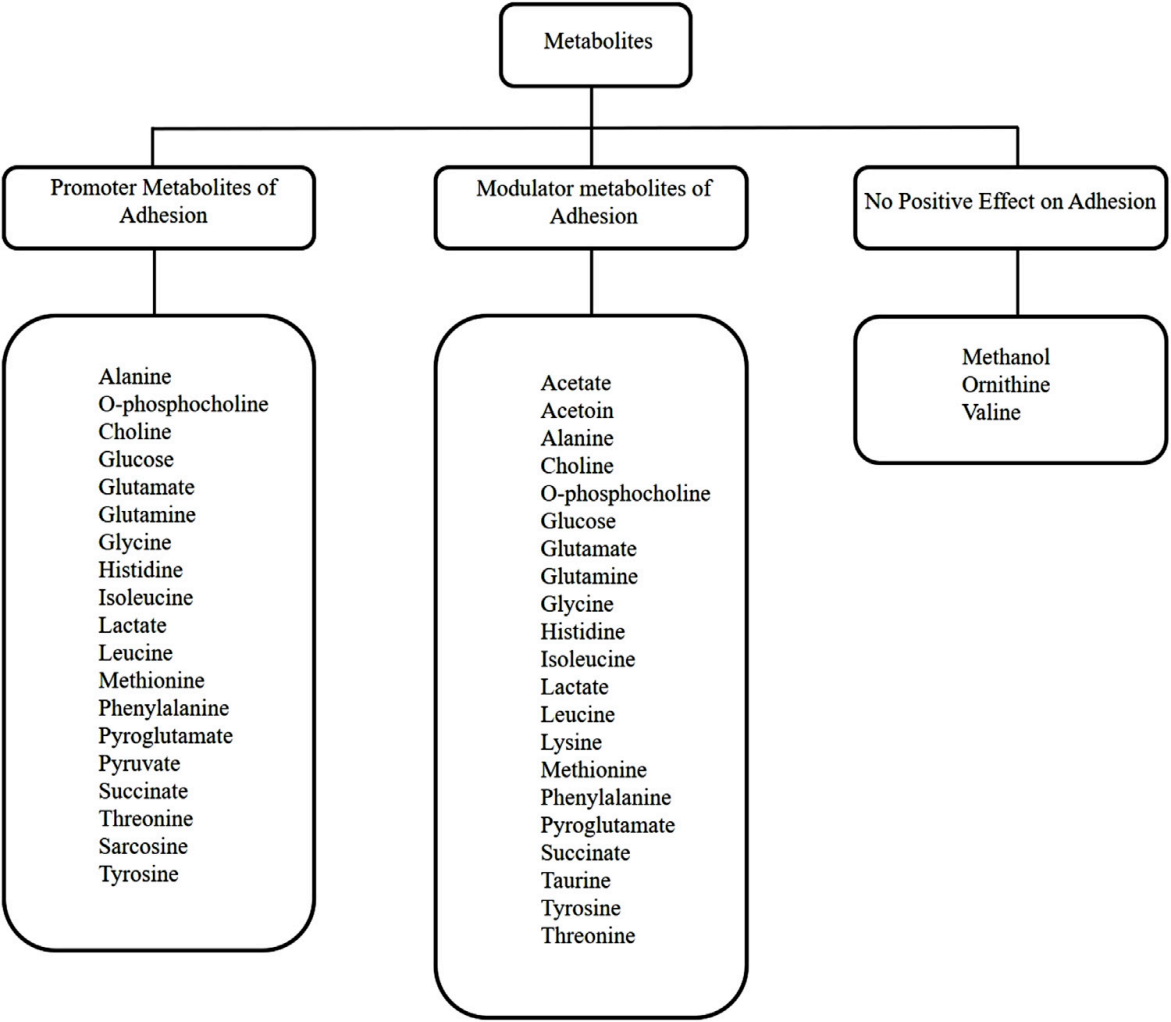
Metabolic pathway diagram summarizing how the twenty-six identified metabolites relate to adhesion and proliferation.

In terms of promoting cellular adhesion (Figure 9), recent research indicates that while primarily a modulator, alanine-grafted materials directly promote cell adhesion by improving substrate interaction and protein adsorption (Dorm et al., 2022). Phosphocholine enhances adhesion by improving epithelial cell adherence and tight junction assembly, thereby serving as a direct promoter in certain cell types (Li et al., 2021). The metabolism of choline within the cell affects the expression of integrins and the spread of cells (Mathow et al., 2015). Consequently, a well-organized SAM that maintains membrane integrity and fluidity would be associated with improved choline metabolism, which is, in turn, linked to enhanced adhesion. Increased glucose concentrations result in enhanced production of adhesion molecules, hence promoting augmented cell adhesion, especially in endothelial cells (Piconi et al., 2004), This suggests that the cellular response to the SAM may involve a glucose-mediated upregulation of these molecules, which would lead to improved adhesion. Suboptimal physiological levels of glutamate augment integrin-mediated adhesion and chemotactic migration of immune cells, acting as a significant facilitator in both mechanisms (Kerage et al., 2019). Glutamine supplementation increases the function of the epithelial barrier and decreases the expression of inflammation-associated adhesion molecules, thereby promoting adhesion in immune and epithelial cells (Hou et al., 2014). When HEK293T cells come into contact with a pro-adhesive SAM, such as ITO-MPS SAM, they need more novo protein synthesis to make strong adhesion plaques. This depends on the amount of glutamine available (Oppenheimer, 1978). This vitamin has a direct effect on the phenotype of cells by changing how they stick to matrix proteins and grow (Oppenheimer, 1978). Glycine enhances cell-substrate interactions and provides cytoprotection, thereby facilitating the stabilization of adhesions (Rahman et al., 2022). While influencing endothelial cell adhesion, histidine-rich glycoproteins facilitate T cell adherence and morphological changes (Olsen et al., 1996). Isoleucine-containing motifs in cell adhesion molecules function as direct mediators of adhesion, facilitating stable binding to extracellular matrix proteins (Kim et al., 2000; Bachmann et al., 2019). Lactate facilitates cytoskeletal dynamics and migration, thereby improving adhesive functions, particularly in the tumor microenvironment (Han et al., 2023). Leucine-rich proteins enhance cell adhesion through the organization of adhesion complexes and the facilitation of integrin-mediated interactions (Kim et al., 2024). Methionine enhances adhesion through the methylation-dependent activation of pathways involved in adhesion and proliferation (Alam et al., 2015). Modified phenylalanine peptides improve cell adhesion and proliferation, especially in tissue engineering and regenerative medicine (Yamada, 2024). Chemokines with pyroglutamate enhance cell migration and adhesion during inflammatory responses (Kehlen et al., 2017). Pyruvate enhances cell migration and adhesion by supporting energy metabolism and signaling pathways associated with adhesion complexes (Han et al., 2013). Sarcosine increases the expression of genes related to adhesion and promotes angiogenesis, thus aiding cell adhesion in cancer progression (Sudhakaran et al., 2014). Activation of succinate receptors improves endothelial barrier integrity and cellular adhesion during inflammation (Atallah et al., 2024). Directly increasing the assembly and stability of adhesion complexes is the phosphorylation of threonine residues in integrins and focal adhesion proteins (Brown et al., 1998). By use of dynamic adhesion reactions, tyrosine phosphorylation controls the assembly and disassembly of adhesion receptors (Burridge et al., 2006). When HEK293 cells first come in contact with a favorable SAM, integrin receptors on their surface group together and turn on focal adhesion kinase (FAK) and Src family kinases. This causes a lot of tyrosine phosphorylation of proteins that help cells stick together, like paxillin and p130Cas (Hartmann, 2022). This phosphorylation causes focal adhesions to form and grow, connecting the cell’s cytoskeleton to the SAM substrate (Hoock et al., 2019).

In terms of modulating cellular adhesion (Figure 9), recent research indicates that acetate serves as a metabolic immunomodulator, which impacts T cell effector operations and possibly influences anti-tumor immunity via changing integrin-mediated adhesion and adhesion-related gene expression, albeit indirectly through epigenetic pathways of regulation (Miller et al., 2023). Acetoin is a neutral metabolic byproduct that is not directly implicated in the enhancement of adhesion but modulates metabolic gene expression and intracellular pH, which could potentially impact the adhesion and the regulation of migratory genes (Cámara-Almirón et al., 2020). Alanine regulates cell adhesion through intracellular signaling processes (Gatlin et al., 2006). Choline modulates protein function and membrane interactions that are essential for adhesion signaling. O-phosphocholine functions as a modulator by affecting cell surface interactions and immunological recognition (Dixit et al., 2022). Glucose influences the expression of adhesion molecules in elevated glucose conditions, hence modulating adhesion (Baumgartner-Parzer et al., 1995). Glutamate modulates immune cell adhesion by enhancing integrin-mediated T cell attachment and facilitating chemotactic movement, functioning through glutamate receptor signaling pathways that alter adhesion dynamics (Ganor et al., 2003). Glutamine modulates the expression of adhesion molecules and the concentration of chemokine receptors on immune and endothelial cells, thereby significantly regulating immune cell infiltration and inflammatory adhesion processes, although not being an adhesion molecule itself (Hou et al., 2014). Glycine regulates cytoprotective mechanisms and affects signaling pathways associated with cell adhesion and membrane stability; however, it does not function as a key promoter of adhesion (Weinberg et al., 2016). Histidine-rich glycoprotein regulates adhesion and immune cell motility through interactions with integrins and other adhesion receptors, influencing adherence without directly forming adhesive connections (Olsen et al., 1996). Although isoleucine is involved in integrin-mediated pathways and modulates cell growth signals, it predominantly influences cell adhesion indirectly through protein synthesis and signaling, rather than functioning as a direct adhesion promoter (Kamranvar et al., 2022). Lactate regulates the synthesis of adhesion molecules and cellular responses in the tumor microenvironment by influencing the activity of immune and stromal cells through intracellular signaling pathways (Llibre et al., 2025). Leucine modulates integrin-mediated adhesion and cellular signaling pathways that affect adhesion dynamics, principally via leucine-rich repeat proteins that organize synaptic and cell-cell adhesion networks (Kim et al., 2024). Lysine modulates cell adhesion via affecting integrin activity and post-translational modifications such as acetylation, which influence adhesion-independent growth and molecular interactions at adhesion sites (Hurd et al., 2023). Thiol groups enhance kidney cell adhesion through redox-mediated signaling, direct cross-linking, and interactions with the extracellular matrix (ECM). This process is largely driven by the versatile chemistry of the sulfhydryl (-SH) group found in the amino acid cysteine (Le-Vinh et al., 2023). Methanol impedes cell-cell adhesion and alters the activities of adhesion molecules by engaging with hydrophobic recognition sites; thus, it serves as a negative modulator of adhesion in specific situations (Wilkemeyer et al., 2000). Methionine functions as a modulator through epigenetic control and methylation processes, affecting the expression of genes associated with adhesion and proliferation without directly facilitating adhesion (N. Zhang, 2018). Phenylalanine primarily functions as a modulator by influencing integrin-mediated adhesion signaling and receptor clustering; increased amounts can disrupt neural adhesion molecules (Hartwig et al., 2006; Kwon et al., 2015). Pyroglutamate influences adhesion by facilitating chemokine maturation and receptor binding, therefore indirectly affecting cell migration and adhesion (Kehlen et al., 2017). Succinate functions as a signaling molecule, activating receptors associated with inflammatory adhesion responses and modifications in endothelial function (Atallah et al., 2024). Taurine regulates intracellular Ca^2+^ homeostasis and affects gene expression associated with adhesion and survival, thereby improving cell adhesion, particularly in endothelial cells (Idrissi et al., 2003). Threonine primarily regulates adhesion dynamics by phosphorylating adhesion-related proteins, including integrins and paxillin, which affects the assembly and disassembly of focal adhesions (M. C. Brown et al., 1998). This means that the surface features of ITO-MPS SAM might affect the activation of certain kinases that phosphorylate threonine residues, which can fine-tune the adhesion response for HEK293 cells. Tyrosine phosphorylation is a key regulator of cell adhesion, influencing integrin activation, signaling kinases, and the aggregation of adhesion receptors (Lin et al., 1994).

Out of the 27 significant metabolites observed, glucose, amino acids (alanine, glutamate, glutamine, glycine, isoleucine, leucine, lysine, methionine, phenylalanine, threonine, tyrosine, valine, and histidine), choline, and o-phosphocholine are potential facilitators for adhesion of HEK293T cells when they are present at high concentration. In contrast, sarcosine, lactate, and pyruvate are potential facilitators for adhesion at low concentrations. Glucose is used to power cellular functions such as ATP generation, which is vital for cytoskeletal remodeling and integrin function. Both of these processes are necessary for adhesion (W-M Fan, 1996). Amongst the metabolites, the amino acids serve as the building blocks for adhesive proteins such as vitronectin and fibronectin, which facilitate the link between cells and the substrate. For example, arginine and lysine are specific amino acids that play crucial roles in cell-substrate interactions (Hynes, 2002; Smallridge, 2004). The other metabolites, O-Phosphocholine and Choline, serve as precursors for phospholipids, which are important components of cell membranes and play a direct role in adhesion by interacting with the substrate and extracellular matrix (ECM) proteins (Vinod et al., 2014). Lactate, another metabolite, functions as a signaling molecule that activates adhesion and integrin activity via G protein-coupled receptors, which are located on the cell surface and are responsible for activating cellular responses and detecting molecules outside the cell. Yang et al. have shown that reduced lactate levels may enhance this signaling more efficiently (Yang et al., 2012). Pyruvate is another potential signaling molecule and metabolite that might influence adhesion at low concentrations. The processes via which it does so are not yet fully understood (Wallace et al., 2014). Sarcosine, yet another metabolite, has the capacity to stimulate p38 MAPK signaling under certain conditions, which in turn may lead to an increase in integrin expression and improved adhesion (Gokhin et al., 2012).

NMR metabolomics has been utilized to investigate cellular metabolism, employing diverse supports and substrates such as tissue culture plastic, hydrogels, fibrous meshes, and many other biomaterials, frequently concentrating on cellular differentiation, proliferation, or responses to certain stimuli (Zhi Hu, 2016). These studies give us an understanding of the metabolomic changes observed in the different substrates, but our findings with SAM-modified ITO represent an advancement in the understanding of metabolite-mediated adhesion, enabled by the precise control afforded by the SAM scaffold. Many laboratories utilize conventional cell culture substrates such as tissue culture polystyrene (TCPS), and their metabolomic profiles provide baseline comparisons. However, less defined and potentially inconsistent cellular microenvironments are observed because TCPS surfaces depend mostly on non-specific protein adsorption for cell attachment (Luk et al., 2000). Our research shows that SAM-modified ITO can provide a more controllable and adaptive surface. The precise manipulation of surface properties, such as hydrophilicity, charge, and functional groups, by SAM chemistry on ITO enables a mechanistic understanding of how these surface traits directly influence cellular metabolism related to adhesion. Metabolomics studies on cells cultured in 3D hydrogels often show altered glucose and lactate metabolism due to diffusion limitations within the scaffold or hypoxia in the core of the construct (Knitsch et al., 2021). Cells on nanofibrous scaffolds may exhibit varying metabolic requirements contingent upon their migratory behaviors, frequently facilitated by glycolytic flow (Reyes-Oliveras et al., 2024). Our study, focused on a 2D altered surface, enables the precise analysis of metabolic changes generated exclusively by surface chemistry, in contrast to the limitations of mass transfer inherent in 3D scaffolds. Employing SAMs rather than 3D scaffolds enables the examination of a clear correlation between surface properties and metabolic processes linked to adhesion (Cox et al., 2002), as the environment is controlled. The growth of cells atop biocompatible plastics or hydrogels can result in significant alterations in lipid metabolism as the cells acclimate to varying surface chemistries or stiffness levels (Lamanna et al., 1999). Our research elucidates the correlation between alterations in choline metabolism and specific surface-induced membrane modifications essential for sustained adhesion, achieved through the application of SAMs to modify molecular surfaces. We could look into how surface energy and charge affect the fluidity of membranes and the appearance of adhesion receptors, since self-assembled monolayers allow for precise control.

### 3.7 Metabolomics data analysis by principal component analysis (PCA)

**FIGURE 10:**
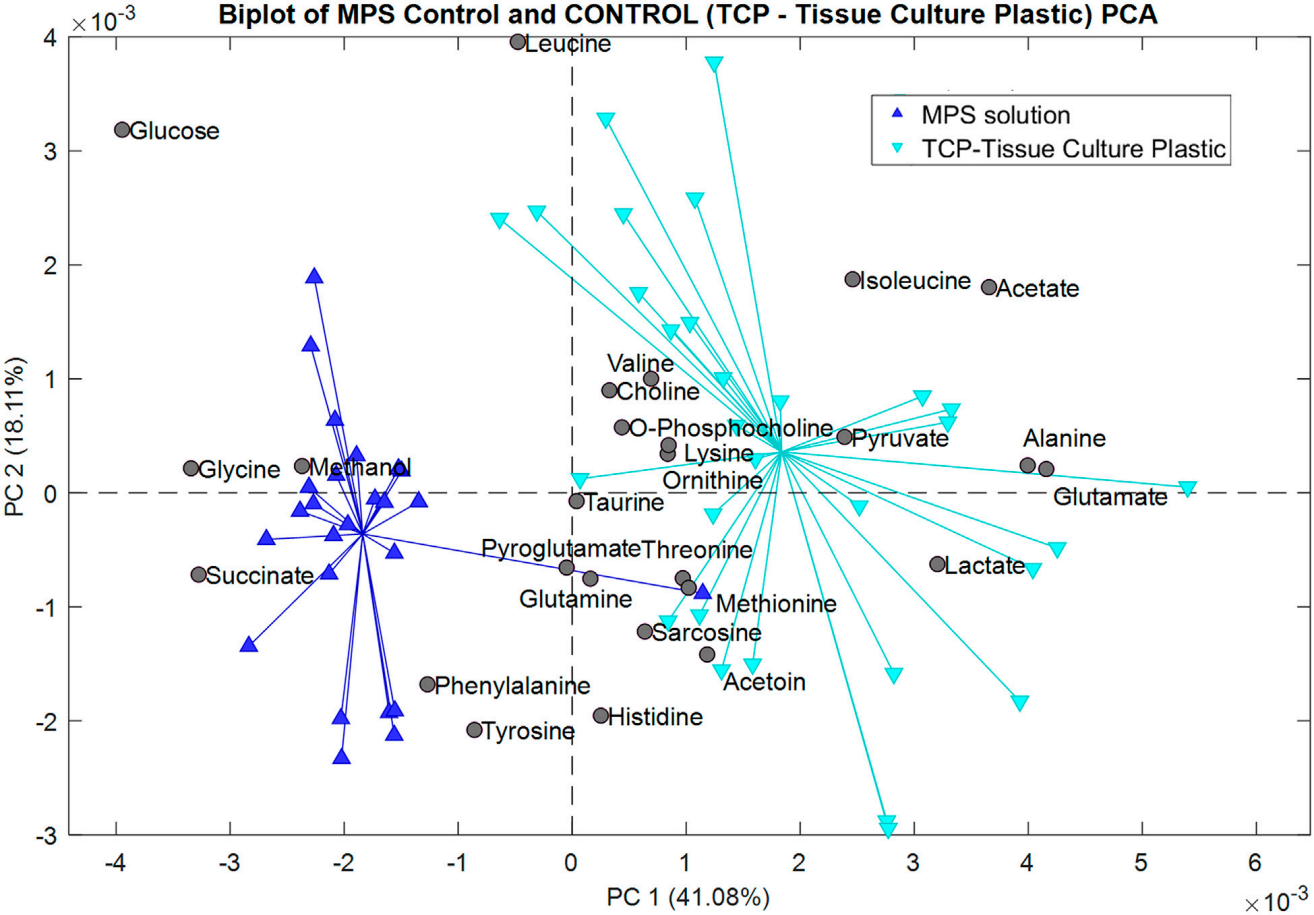
Biplot showing the principal component analysis of the MPS solution control compared to the Tissue Culture Plastic (TCP).

A metabolomics analysis of the data was performed using principal component analysis (PCA) with a PLS Toolbox (Eigenvector Research, Inc.) in MATLAB (MathWorks, LLC). PCA was initially used to reduce the dimensionality into a smaller set of variables while preserving the information provided by the larger set of data. PCA was also used for data visualization to drown out the noise and reveal any significant patterns (Kurita, 2021). Each model compared a class of data against the same control group, the MPS solution. The control group lacked the ITO-MPS and the ITO substrate and contained only the MPS solution. The generated models were based on both the mean center and Pareto scaling. The following plots represent the scores of the second principal component (PC2) *versus* the scores of the first principal component (PC1) analysis. The plots also illustrate the contribution of each metabolite to the different classes of data. Figure 10 shows that the following upregulated metabolites have the.

**FIGURE 11:**
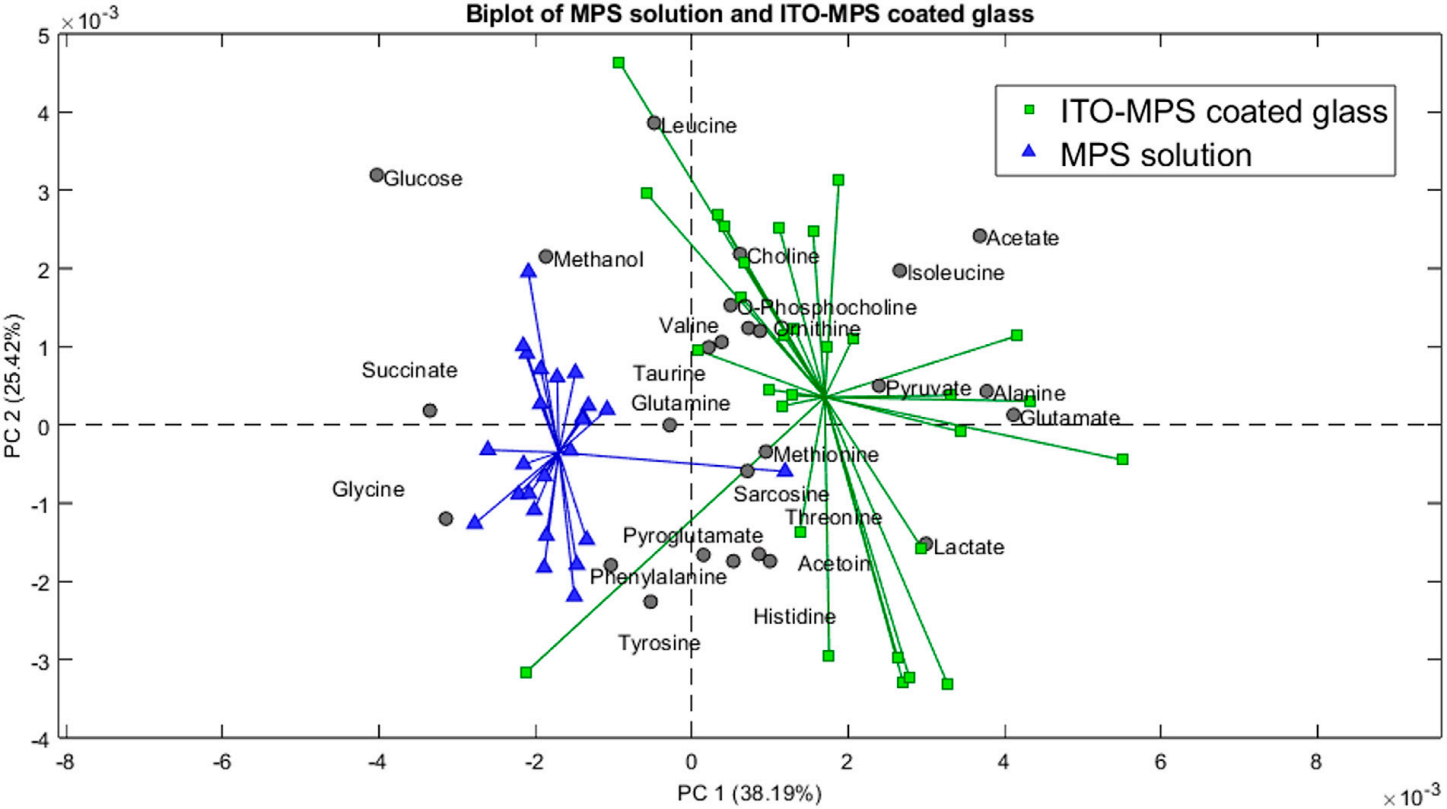
Biplot showing the principal component analysis of the MPS solution compared to the ITO-MPS coated glass.

Greatest contribution in the positive field: lactate, pyruvate, glutamate, acetate, alanine, and isoleucine. These metabolites are barely present, and in some cases, totally absent from the samples containing the negative control (MPS solution), but were present in the samples cultured on the TCP substrate, indicating that these metabolites were utilized by the cells during growth. Additionally, the following downregulated metabolites have the greatest contribution in the negative field: glucose, glycine, methanol, and succinate, since they were more present in the negative control than in the TCP, showing that they were not being utilized by the cells since the cells could not survive in such conditions for a long time. In Figure 11, we compared the metabolomic profiles of the negative.

**FIGURE 12:**
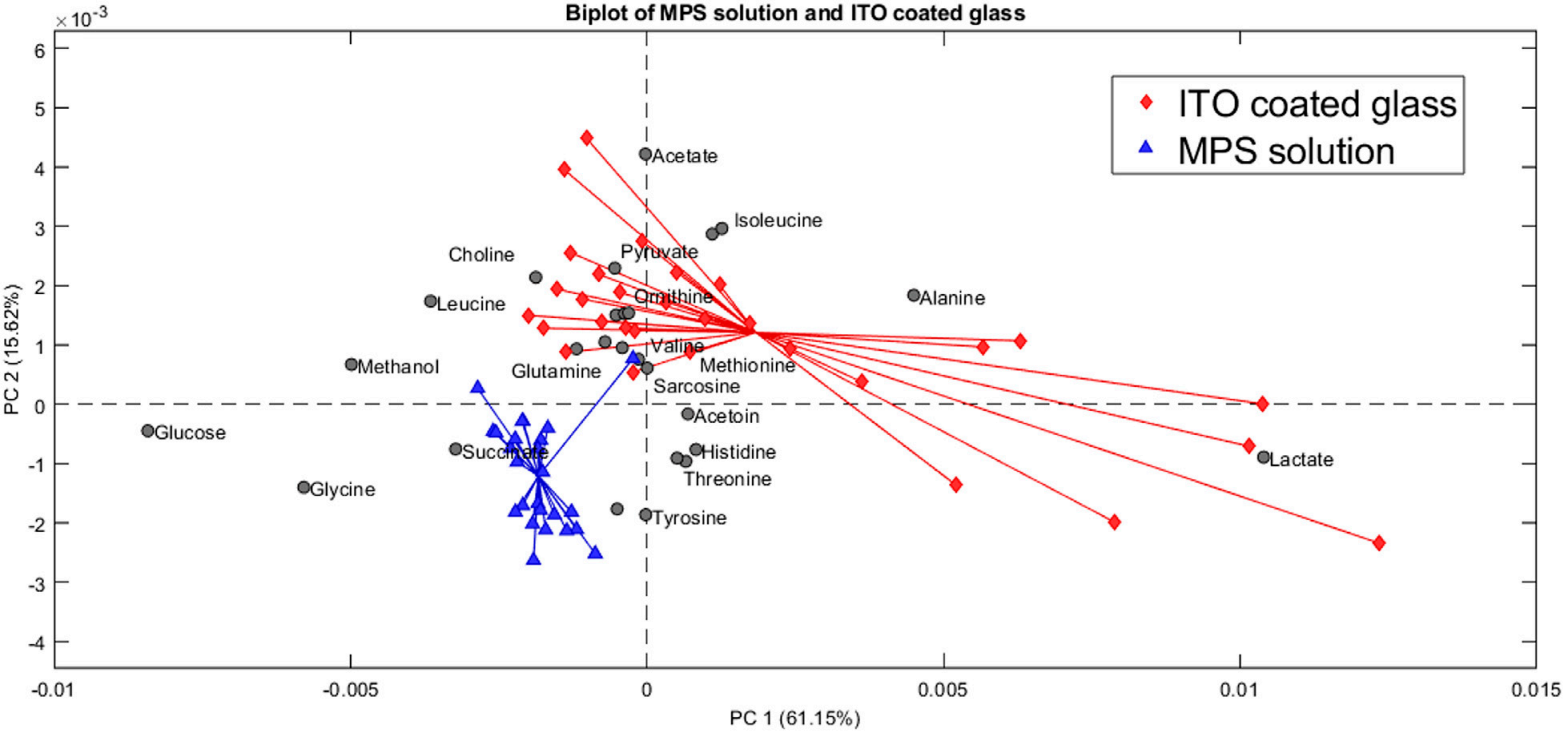
Biplot showing the principal component analysis of the MPS solution compared to the ITO-coated glass.

Control to that of the ITO-MPS SAM-coated substrate, and we observe that the following upregulated metabolites have the greatest contribution in the positive field: lactate, pyruvate, glutamate, acetate, alanine, and isoleucine. Additionally, the following downregulated metabolites have the greatest contribution in the negative field: glucose, glycine, methanol, and succinate. We observe that the metabolites that contributed to the positive field were similar to those that contributed to the positive field in the TCP, which is the regular cell culture vessel. Figure 12 shows the metabolites contributing to the positive field in the ITO substrate compared to the negative control. We observe that the following upregulated metabolites had the greatest contribution in the positive field: lactate, pyruvate, glutamate, acetate, alanine, and isoleucine. Additionally, the following downregulated metabolites have the greatest contribution in the negative field: glucose, glycine, methanol, and succinate. From our experiments, we notice that the ITO-SAM, ITO-MPS SAM-coated substrate, and the TCP contribute to the growth and proliferation of the cells. This is confirmed by the presence of the same.

Metabolites contributing to the positive field in these three substrates, with an abundance of these metabolites found on the ITO-MPS SAM-coated substrate. The observed upregulation and downregulation of these metabolites can provide key information related to disease progression, drug metabolism, and the development of potential new therapies. A statistical t-test was also performed for the metabolites in Excel. The purpose of this test is to compare the difference in the variance in the means. Each metabolite passed the t-test with a P value less than 0.05. From the results obtained from the PCA plots, we can conclude that the upregulated metabolites were abundant in the ITO-MPS SAM-Coated substrate, followed by the TCP, which is the positive control. These upregulated metabolites played a role in promoting the adhesion of the cells. We also see that the downregulated metabolites were abundant in the samples containing MPS, which was the negative control. These metabolites played a role in preventing cellular adhesion and eventually led to cell death.

### 3.8 Mechanistic insight into the modulation of the cellular microenvironment

3-(Mercaptopropyl) trimethoxysilane (MPS) has a thiol (-SH) functional group and trimethoxysilane groups (Kobayashi et al., 2016a). Trimethoxysilane groups can connect strongly with the hydroxyl groups on the ITO surface. Thus, a strong and steady self-assembled monolayer (SAM) is created (Choi and Noh, 2008). The most important thing about the MPS change is that it has reactive thiol (-SH) groups on the surface (Kobayashi et al., 2016b). These thiol groups can be further modified with different biomolecules, such as proteins, peptides, and polymers, by using thiol-ene click chemistry or making disulfide bonds (Zhang et al., 2001). This tunable functionalization is the key to controlling the surroundings of cells (Shah et al., 2009). There are reactive thiol groups in MPS that can exactly immobilize Arg-Gly-Asp (RGD) peptides, collagen, fibronectin, and other desired proteins (Shah et al., 2009). By covalent attachment of certain cell-adhesive proteins to the MPS layer, researchers can produce micropatterned surfaces that direct cell attachment in a very specific way (Shah et al., 2009). For example, Collagen I has been able to stick well to ITO surfaces to create patterns for cell attachment (Shah et al., 2009). This adsorption gives cells important molecular cues that change how they behave. The MPS layer has a direct effect on integrin activation by making it easier for certain integrin-binding ligands (like collagen or RGD peptides) to stay in place (Shah et al., 2009). Integrins can recognize certain amino acid patterns in ECM proteins (Luo et al., 2007). According to Luo et al. (2007), these ligands can start “outside-in” signaling, which changes the shape of integrins and turns them on, as long as they are placed in a certain way on the MPS-based surface. ITO is a material that is electrochemically active (Shah et al., 2009). Even though MPS is usually used for chemical functionalization, some studies have looked at how electric fields change the way cells interact with the ITO surface. If the MPS layer enables regulated charge transfer or electrical stimulation, integrin activity may be modulated since electrical stimuli can modify cellular adhesion and communication (Shah et al., 2009).

### 3.9 Organoid formation implications

Organoids are tiny, three-dimensional (3D) structures grown in a lab from stem cells or organ progenitors that mimic the shape and function of real organs. This makes them very important for studying how humans develop and get sick (Lancaster and Knoblich, 2014). You can utilize organoid culture media to improve metabolites that help cells stick together, like glucose, glutamine, and poly-L-lysine (PLL). This will help cells group together at first and then organize themselves later (Lancaster and Knoblich, 2014). For example, glucose is a very important source of energy, and its levels are carefully controlled to produce adhesion molecules. These molecules are necessary for the structural stability and dense arrangement observed in complex organoid structures (Morales and Andrews, 2022). Altering metabolites such as acetate and lactate can influence cell fate determinations and spatial arrangement within organoids. Acetate’s role in histone acetylation might help some types of cells grow faster. These cells need particular adhesive properties to stay in place inside the growing organoid (Lancaster and Knoblich, 2014). In the same manner, regulating lactate levels might let organoids grow on their own, which would change how cells move and how the cytoskeleton works. This ensures that cells are organized properly and that correct tissues form.

### 3.10 Applications in regenerative medicine

How metabolites assist organoids to adhere together has a direct effect on regenerative medicine. This is especially true when it comes to designing and testing medications that work for each person. One can manufacture organoids from Induced Pluripotent Stem Cells (iPSCs) collected from people. This is an excellent approach to learn about human diseases, such as cancer and metabolic disorders (Lancaster and Knoblich, 2014). Pyruvate is an important aspect of how the body uses energy. It can also be altered to examine metabolic alterations occurring in diseases that impair cellular adhesion, such as cancer progression, where these modifications are prevalent (Wrzesinski and Fey, 2018). Liver organoids are a wonderful tool to test medications that modify how the human liver operates because they have their own metabolism (Lancaster and Knoblich, 2014). Intestinal organoids are an excellent approach to learn more about how medications operate, where they go, and how bad they are for the gut (Kourula et al., 2023). Metabolites are highly crucial for generating tissues that can be transplanted because they help cells stick together. Biomaterial scaffolds may be able to enable transplanted cells or organoids to link with host organs by adding poly-L-lysine and poly-L-ornithine to them. These improvements would fix several problems with how organ transplants are done presently, such as problems with the immune system and rejection (Lancaster and Knoblich, 2014). Researchers might be able to make sure that altered tissues stick together successfully by making the optimum metabolic environment possible. After being transplanted, this will assist the cells in staying alive and performing their function better. In personalized medicine, patient-derived organoids (PDOs) enable physicians to evaluate the efficacy of pharmaceuticals on tissues that precisely reflect the patient’s genetic and phenotypic characteristics (Lancaster and Knoblich, 2014). If one knows how each patient’s specific metabolite profiles affect the binding in their PDOs, one might be able to make treatments work better.

## 4 Conclusion

The human embryonic kidney 293T (HEK293T) cell is commonly used by researchers for cell biological studies because of its many advantages. The method of enhancing HEK293T cell adhesion and proliferation using a SAM scaffold, as discussed here, has not been reported before. This study also illustrates the extensive potential of surface modification techniques, particularly the use of SAMs, in optimizing substrates for *in vitro* cell cultivation. The successful implementation of ITO-MPS SAM-coated substrates for HEK293T cells shows that the physicochemical characteristics of culture surfaces can substantially enhance cellular behavior. The cell proliferation on the SAM scaffolds was quantified using the MTT assay, while confocal microscopy images were utilized to validate the findings. To our knowledge, the NMR metabolomics for HEK293T cells on a SAM scaffold has not been reported before. ^1^H NMR spectroscopy was employed to examine the metabolomics in the media with and without ITO-MPS-SAM coated substrates for a duration of 120 h. The MTT assay results indicate that the ITO-MPS SAM-coated substrate promotes increased cell adherence and proliferation. The visual validation of the enhanced cellular environment is provided by the confocal microscopy images, which corroborate these findings. The biochemical changes associated with enhanced cellular adhesion were further illuminated by the metabolomic analysis of cell cultures on these modified surfaces. Twenty-six metabolites were identified in the metabolomic analysis. Sixteen metabolites were identified as enhancing and modulating adhesion; sixteen of which are both modulator and promoter metabolites of adhesion. These include alanine, phosphocholine, glucose, glutamate, glutamine, glycine, histidine, isoleucine, lactate, leucine, methionine, phenylalanine, pyroglutamate, succinate, threonine, and tyrosine. These results suggest that these metabolites have a significant role to play in improving the cellular adhesion beyond the known period of 72 h. The results indicate that the ITO-MPS SAM scaffold improved the cellular environment, actively influenced cellular metabolic pathways, thereby facilitating adhesion and proliferation. The identification of specific metabolites that are associated with adhesion provides valuable insights for future research on the influence of metabolic control on cell-substrate interactions, which may be particularly useful in the fields of regenerative medicine and tissue engineering. Due to the rising need for reliable and accurate *in vitro* models in drug discovery, tissue engineering, and organoid research, this method offers a potential strategy for enhancing cell adhesion and proliferation in diverse cell types. In summary, the findings from this study represent a significant advancement in the optimization of cell culture conditions for HEK293T cells. The improved adhesion and proliferation noted on ITO-MPS SAM-coated substrates, along with the acquired metabolomic insights, provide a novel perspective on the interaction between surface chemistry and cellular metabolism in affecting cell behavior.

## Data Availability

The datasets generated and analyzed for this study can be found at https://data.mendeley.com/datasets/5xfz78zwsr/1.
