# Supplementary material for: Investigation of enhanced adhesion of HEK293T cells on SAM-modified ITO surfaces using NMR metabolomics
Source: Front Bioeng Biotechnol. 2025 Sep 25;13:1652675. doi: 10.3389/fbioe.2025.1652675 (PMC12507750; doi:10.3389/fbioe.2025.1652675)
Supplement: Supplementary file 1 [file Supplementaryfile1.docx]

Supplementary Material

**TABLE OF CONTENTS**

**List of Supporting Figures**

**Supplementary Figure 1.** The FTIR/ATR spectrum of ITO-MPS on a glass substrate.

**Supplementary Figure 2.** MTT assay showing the absorbance of HEK 293T cells cultured on ITO, ITO-APTES, ITO-ODT, and ITO-MPS SAM-coated glass substrates for a period of 12 days.

**Supplementary Figure 3.** The Bradford assay showing total protein concentration of HEK 293T cells cultured on ITO, ITO-APTES, ITO-ODT, and ITO-MPS SAM-coated glass substrates for a period of 12 days.

**Supplementary Figure 4.** Confocal Microscope images of HEK 293T cells on a. ITO b. ITO-APTES c. ITO-ODT and d. ITO-MPS SAM coated substrates (10x) after 120 hours of culture.

**Supplementary Figure 5.** Confocal Microscope images of HEK 293T cells cultured on ITO, ITO-MPS, MPS, TCPs (10x) from Day 1 (24 hrs) of culture to Day 5 (120 hrs) of culture.

**Supplementary Figure 6.** Confocal Microscope images of HEK 293T cells cultured on ITO-MPS with magnifications of a. 4x b. 10x c. 20x d. 40x after 120 hours of culture.

**List of Supporting Tables**

**Table S1.** Metabolites significantly observed in the aqueous extract of HEK 293T Cell Cultured Media, and their proton chemical shifts. Student t tests were applied, and a False Discovery Rate (FDR) was applied for multiple testing correction.

**
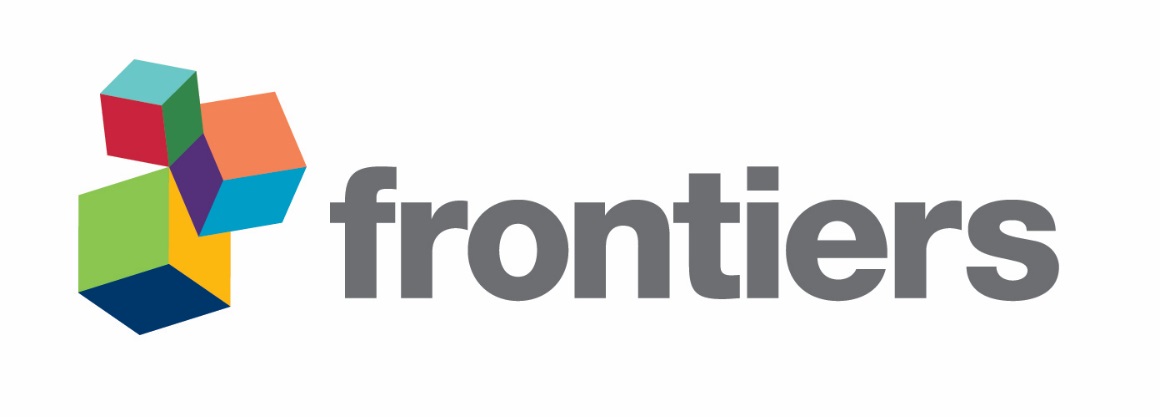
**


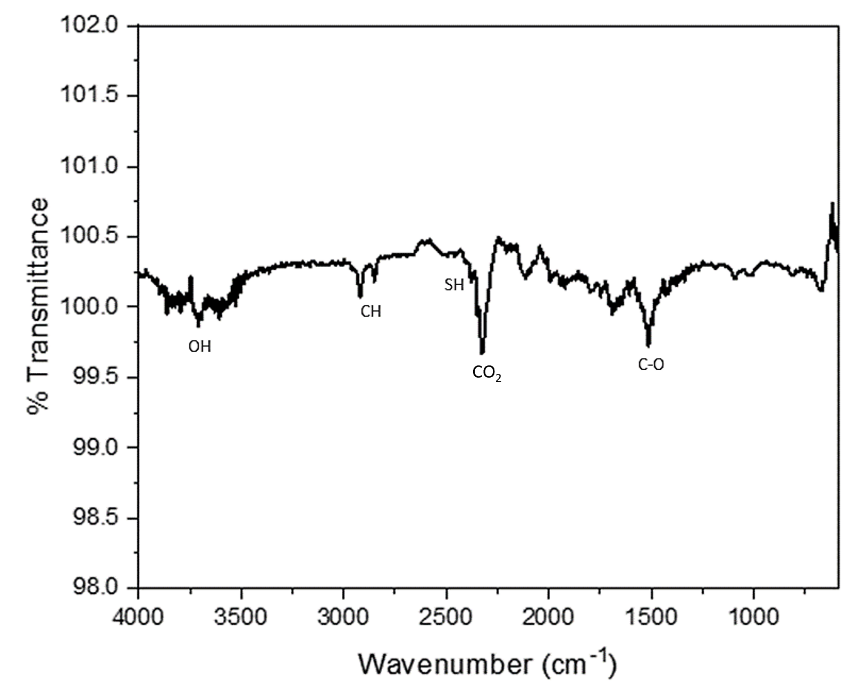


**Supplementary Figure 1.** The FTIR/ATR spectrum of ITO-MPS on a glass substrate.


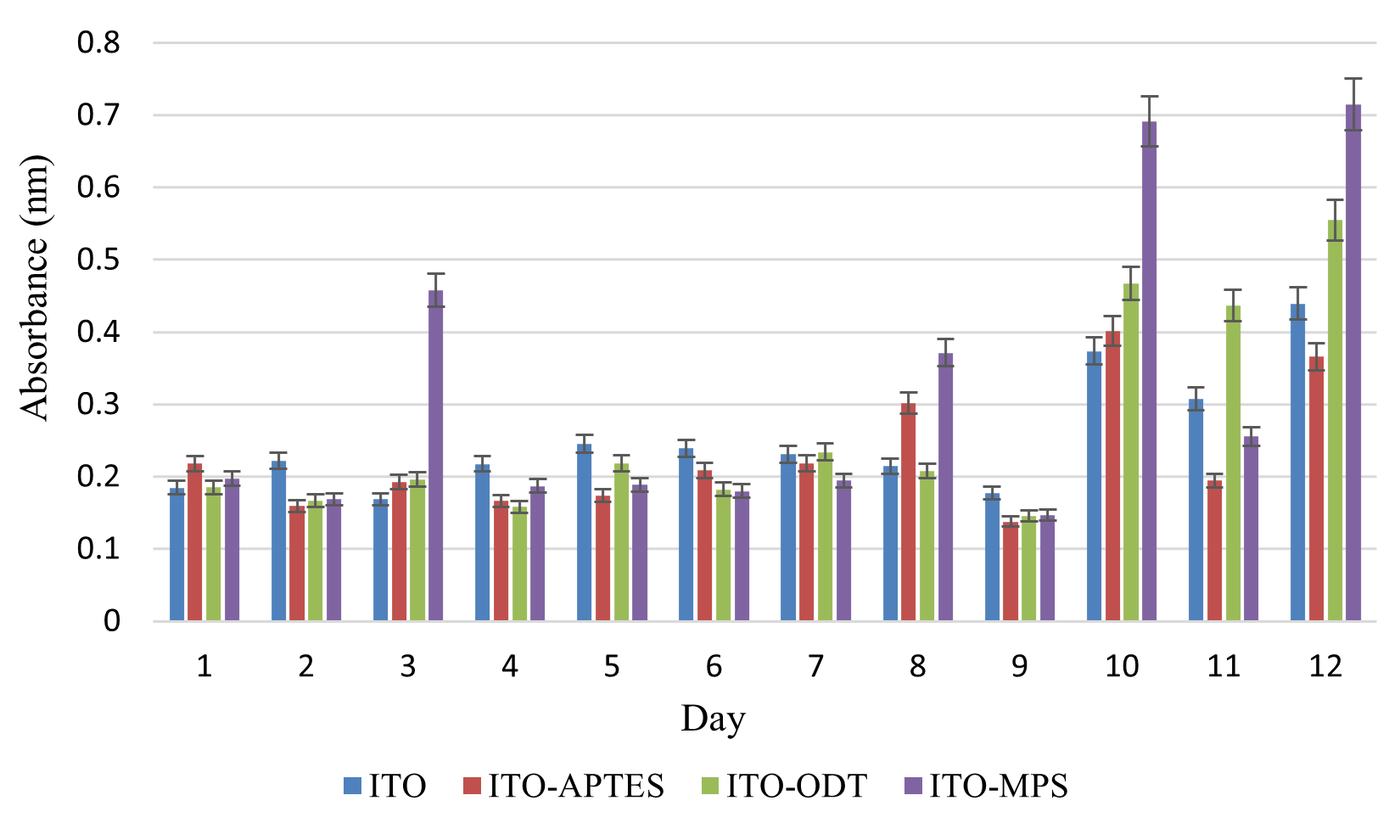


**Supplementary Figure 2** MTT assay showing the absorbance of HEK 293T cells cultured on ITO, ITO-APTES, ITO-ODT and ITO-MPS SAM-coated glass substrates for a period of 12 days.


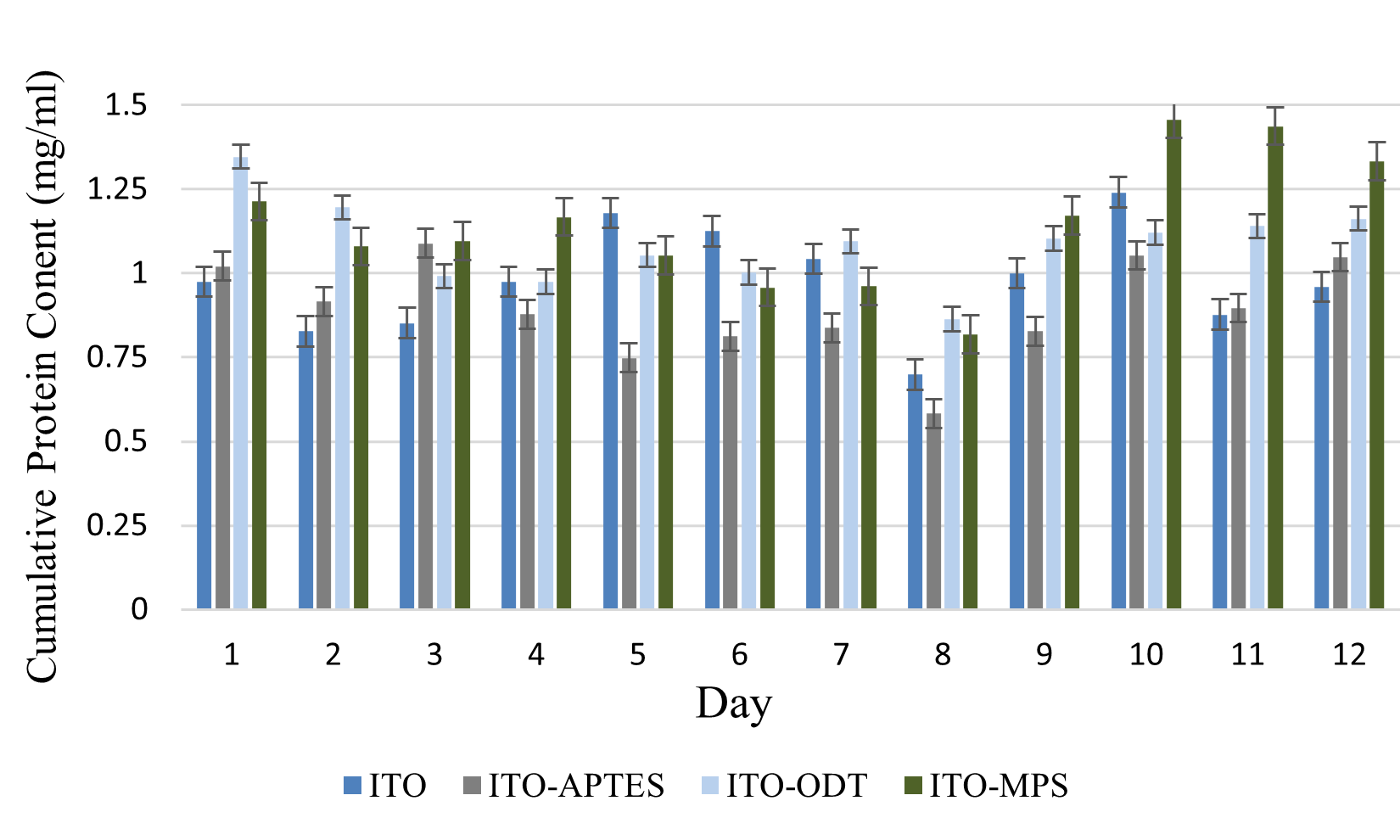


**Supplementary Figure 3.** The Bradford assay showing total protein concentration of HEK 293T cells cultured on ITO, ITO-APTES, ITO-ODT and ITO-MPS SAM-coated glass substrates for a period of 12 days.


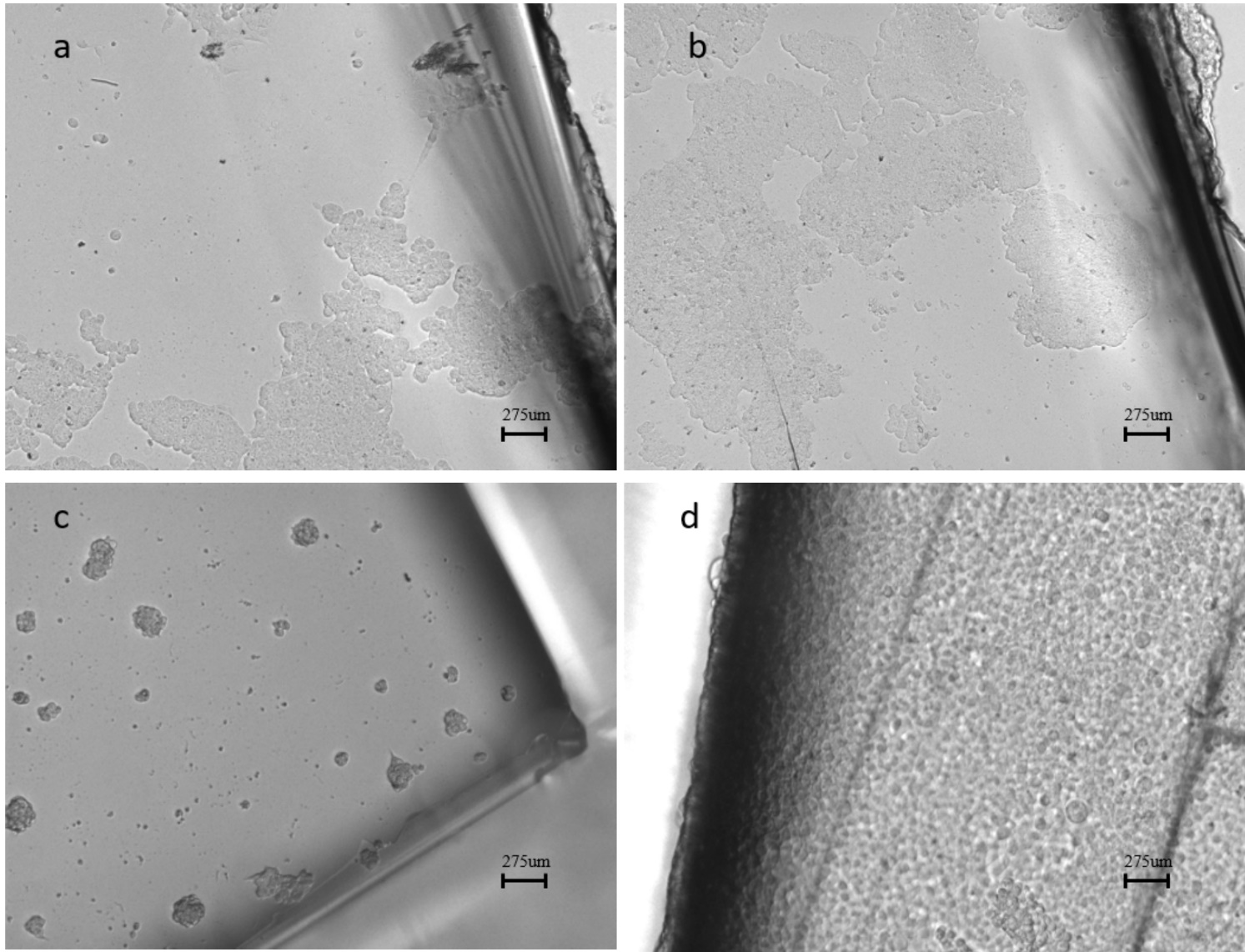


**Supplementary Figure 4.** Confocal Microscope images of HEK 293T cells on a. ITO b. ITO-APTES c. ITO-ODT and d. ITO-MPS SAM coated substrates (x10) after 120 hours of culture.


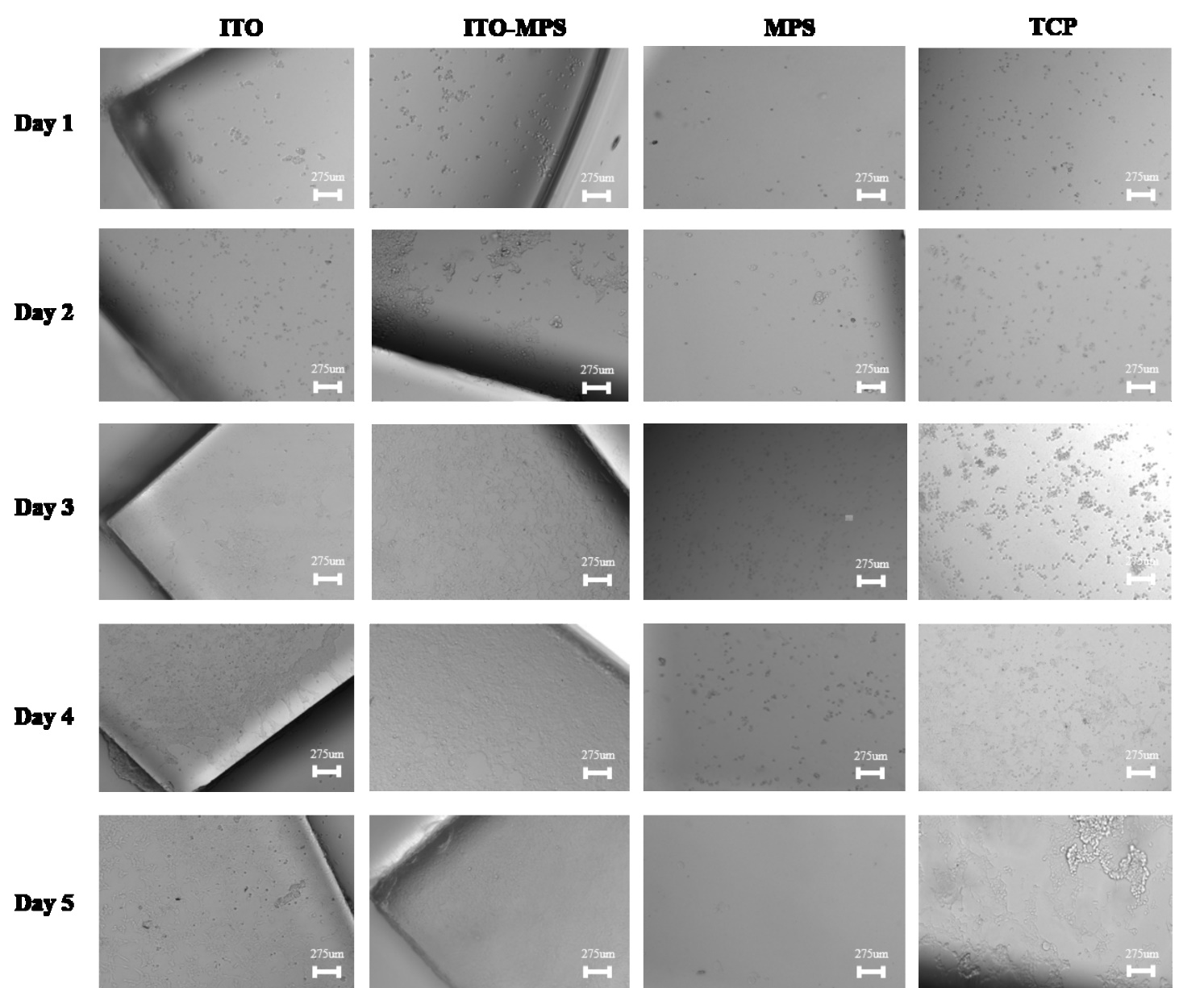


**Supplementary Figure 5.** Confocal Microscope images of HEK 293T cells cultured on ITO, ITO-MPS, MPS, TCPs (10x) from Day 1 (24 hrs) of culture to Day 5 (120 hrs) of culture.


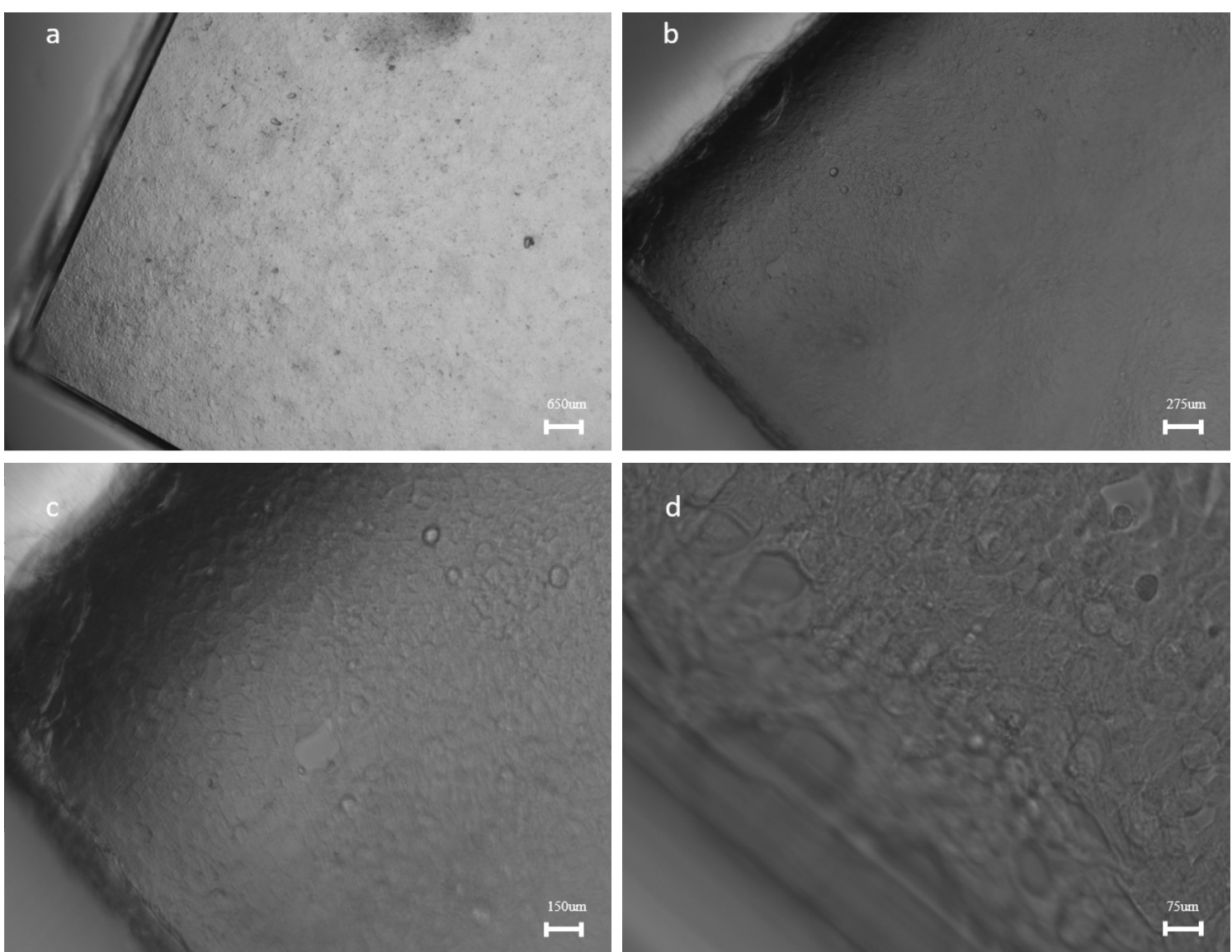


**Supplementary Figure 6.** Confocal Microscope images of HEK 293T cells cultured on ITO-MPS with magnifications of a. 4x b. 10x c. 20x d. 40x after120 hours of culture.

**Supplementary Table 1**: Metabolites significantly observed in the aqueous extract of HEK 293T Cell Cultured Media, and their proton chemical shifts. Student t tests were applied and a False Discovery Rate (FDR) was applied for multiple testing correction.

|  |  | Group 1 vs Control | | | Group 2 vs Control | | | Group 4 vs Control | | |
| --- | --- | --- | --- | --- | --- | --- | --- | --- | --- | --- |
|  | ppm | t_test | Fold Change | FDR Q | t_test | Fold Change | FDR Q | t_test | Fold Change | FDR Q |
| Acetate | 1.92 | 9.65E-10 | 1.16 | 5.02E-09 | 8.57E-14 | 1.20 | 7.43E-13 | 6.10E-18 | 1.21 | 1.59E-16 |
| Alanine | 1.47 | 4.66E-15 | 1.50 | 1.21E-13 | 8.03E-15 | 1.31 | 2.09E-13 | 1.31E-16 | 1.35 | 1.71E-15 |
| Glutamate | 2.33 | 3.02E-14 | 2.30 | 3.93E-13 | 5.10E-14 | 2.80 | 6.63E-13 | 1.78E-14 | 2.89 | 1.54E-13 |
| Glycine | 3.56 | 2.73E-08 | 0.81 | 1.18E-07 | 2.88E-07 | 0.92 | 1.25E-06 | 2.29E-11 | 0.90 | 1.49E-10 |
| Phenylalanine | 7.35 | 4.11E-12 | 0.76 | 3.56E-11 | 8.39E-09 | 0.78 | 4.37E-08 | 2.97E-10 | 0.77 | 1.54E-09 |
| Isoleucine | 0.94 | 1.46E-11 | 1.23 | 9.46E-11 | 1.50E-09 | 1.23 | 9.73E-09 | 1.27E-09 | 1.21 | 5.52E-09 |
| Methanol | 3.38 | 6.72E-05 | 0.78 | 1.75E-04 | 3.44E-03 | 0.91 | 7.45E-03 | 4.57E-06 | 0.88 | 1.70E-05 |
| Pyruvate | 2.38 | 1.48E-03 | 1.08 | 3.20E-03 | 3.43E-05 | 1.11 | 1.11E-04 | 7.47E-06 | 1.12 | 2.43E-05 |
| Succinate | 2.45 | 2.05E-05 | 0.79 | 5.91E-05 | 7.51E-06 | 0.77 | 2.79E-05 | 1.58E-05 | 0.77 | 4.57E-05 |
| Glucose | 3.74 | 5.62E-06 | 0.80 | 1.83E-05 | 6.09E-05 | 0.92 | 1.76E-04 | 2.43E-05 | 0.92 | 6.31E-05 |
| Tyrosine | 7.21 | 8.13E-07 | 0.71 | 3.02E-06 | 5.75E-04 | 0.78 | 1.36E-03 | 7.47E-05 | 0.77 | 1.76E-04 |
| Lactate | 4.12 | 2.30E-04 | 3.02 | 5.44E-04 | 9.05E-05 | 1.30 | 2.35E-04 | 2.58E-04 | 1.36 | 5.58E-04 |
| Ornithine | 3.07 | 9.65E-03 | 1.17 | 1.93E-02 | 5.93E-03 | 1.19 | 1.19E-02 | 8.82E-04 | 1.22 | 1.76E-03 |
| Acetoin | 4.44 | 8.14E-02 | 1.50 | 1.32E-01 | 4.93E-02 | 1.63 | 9.15E-02 | 2.00E-03 | 1.82 | 3.72E-03 |
| Methionine | 2.65 | 3.09E-01 | 1.05 | 4.46E-01 | 1.28E-01 | 1.09 | 1.67E-01 | 3.83E-03 | 1.16 | 6.63E-03 |
| Threonine | 4.26 | 8.58E-01 | 0.99 | 8.93E-01 | 6.69E-02 | 1.12 | 1.09E-01 | 5.29E-03 | 1.15 | 8.60E-03 |
| Lysine | 3.03 | 5.16E-02 | 1.04 | 9.59E-02 | 5.26E-02 | 1.04 | 9.12E-02 | 7.45E-03 | 1.04 | 1.14E-02 |
| Valine | 1.04 | 8.80E-02 | 1.03 | 1.35E-01 | 6.90E-02 | 1.03 | 1.06E-01 | 1.41E-02 | 1.03 | 2.03E-02 |
| O-Phosphocholine | 3.19 | 7.11E-02 | 1.17 | 1.23E-01 | 9.63E-02 | 1.17 | 1.32E-01 | 4.70E-02 | 1.17 | 6.43E-02 |
| Leucine | 0.96 | 4.50E-01 | 0.99 | 5.32E-01 | 1.79E-01 | 1.02 | 2.21E-01 | 1.34E-01 | 1.02 | 1.74E-01 |
| Choline | 3.21 | 8.89E-01 | 1.01 | 8.89E-01 | 9.59E-02 | 1.10 | 1.38E-01 | 1.37E-01 | 1.08 | 1.69E-01 |
| Glutamine | 2.17 | 4.23E-01 | 1.04 | 5.24E-01 | 6.01E-01 | 1.02 | 6.80E-01 | 3.02E-01 | 1.04 | 3.57E-01 |
| Sarcosine | 2.73 | 7.77E-01 | 1.04 | 8.78E-01 | 9.81E-01 | 1.00 | 9.81E-01 | 4.81E-01 | 1.09 | 5.43E-01 |
| Taurine | 3.32 | 3.49E-01 | 0.93 | 4.78E-01 | 3.81E-01 | 0.93 | 4.50E-01 | 5.73E-01 | 0.96 | 6.21E-01 |
| Histidine | 7.07 | 3.72E-01 | 0.76 | 4.84E-01 | 9.51E-01 | 0.98 | 9.89E-01 | 7.63E-01 | 0.91 | 7.93E-01 |
| Pyroglutamate | 4.18 | 8.17E-01 | 0.98 | 8.85E-01 | 6.59E-01 | 0.98 | 7.14E-01 | 8.83E-01 | 0.99 | 8.83E-01 |
